# *Rhoptry protein 5* (*ROP5*) Is a Key Virulence Factor in *Neospora caninum*

**DOI:** 10.3389/fmicb.2017.00370

**Published:** 2017-03-07

**Authors:** Lei Ma, Jing Liu, Muzi Li, Yong Fu, Xiao Zhang, Qun Liu

**Affiliations:** Key Laboratory of Animal Epidemiology and Zoonosis, Ministry of Agriculture, National Animal Protozoa Laboratory, College of Veterinary Medicine, China Agricultural UniversityBeijing, China

**Keywords:** *Neospora caninum*, gene editing, rhoptry protein 5, virulence factor, protozoan parasites

## Abstract

*Neospora caninum*, of the *Apicomplexa* phylum, is a common cause of abortions in cattle and nervous system dysfunction in dogs. Rhoptry proteins of *Apicomplexa* play an important role in virulence. The objectives of this study were to study functions of NcROP5 in *N. caninum* by deleting the NcROP5 gene from the wild Nc-1 strain. We selected *NcROP5* in ToxoDB and successfully constructed an *NcROP5* gene-deleted vector, pTCR-NcROP5-CD KO. Then we screened the NcROP5 knockout strains (ΔNcROP5) at the gene, protein and transcription levels. Plaque assay, host cell invasion assay and intracellular proliferation test showed that the ΔNcROP5 strain had less plaque space, weakened invasion capacity and slower intracellular growth. Animal testing showed significantly lower cerebral load of ΔNcROP5 than the load of the Nc-1 strain, as well as a loss of virulence for the ΔNcROP5 strains. Phenotypic analyses using the label-free LC-MS/MS assay-based proteomic method and KEGG pathway enrichment analysis showed a reduction of *NcGRA7* transcription and altered expression of multiple proteins including the apicomplexan family of binding proteins. The present study indicated that ROP5 is a key virulence factor in *N. caninum* in mice. The proteomic profiling of Nc-1 and ΔNcROP5 provided some data on differential proteins. These data provide a foundation for future research of protein functions in *N. caninum*.

## Introduction

*Neospora caninum* is a common cause of abortions in cattle and nervous system dysfunction in dogs (Hall et al., 2005; Lyon, 2010). The parasite has become an international concern due to the connection of the parasite infection to abortions in dairy and beef cattle worldwide (Dubey, 1999). Antibodies to *N. caninum* have been detected in humans in Brazil, Korea, Northern Ireland, and the United States, although viable parasites have not been isolated (Dubey et al., 2007). Investigations are ongoing to determine whether the pathogen is a potential threat to human health.

*Neospora caninum* and *Toxoplasmosis gondii* belong to the same Apicomplexa family and share similar morphology, host range and clinical symptoms, although they appear to be divergent in their pathogenicity in mice (Lyon, 2010). A tachyzoite of the *T. gondii* RH strain can lead to death in a mouse (Saeij et al., 2006; Taylor et al., 2006), whereas the same outcome requires more than 10^6^ tachyzoites of the *N. caninum* (Tao et al., 2014; Arranz-Solís et al., 2015). Rhoptry proteins (ROPs), including TgROP5, TgROP16, TgROP17, and TgROP18 are impotant virulence factors of *T. gondii* (TgROPs) (Yamamoto et al., 2009; Etheridge et al., 2014; Behnke et al., 2015; Shwab et al., 2015). Deletion of the *TgROP5* gene, which forms a complex with TgROP18, results in a complete loss of virulence (Reese and Boothroyd, 2011). We hypothesized that the above genes had similar functions in *N. caninum*. Unexpectedly, we found that *ROP18* in *N. caninum* was a pseudogene (Tao et al., 2014). We suspected that another rhoptry gene of *N. caninum* might play a crucial role in virulence. Transgenic strains of *T. gondii* and *Plasmodium falciparum* of *Apicomplexa* parasites have been widely used to study the functions of parasite proteins (Ghorbal et al., 2014; Hui et al., 2014; Shen et al., 2014; Hammoudi et al., 2015; Williams et al., 2015; Li et al., 2016). However, no laboratory strains and genome-editing techniques have been developed and no transgenic strains constructed for the study of protein functions of *N. caninum*. We sought to construct a ROP5-knockout strain of *N. caninum* and determine the function of ROP5 in this parasite.

Firstly, we constructed a recombinant plasmid containing the untranslated region of the *NcROP5* gene and obtained a NcROP5-deficienct strain (ΔNcROP5). The NcROP5-deficienct parasite exhibited, weakened host invasion, proliferation and virulence, compared with the wild parent strain. The technique we developed and the new transgenic strain laid ground work for future research of this highly pathogenic parasite.

## Materials and Methods

### Ethics Statement

The experiments were performed in strict accordance with the recommendations in the Guide for the Care and Use of Laboratory Animals, Ministry of Science and Technology, China. All experimental proceduresw ere approved by the Institutional Animal Care and Use Committee of China Agricultural University (under the certificate of Beijing Laboratory Animal employee ID: 18049). The mice were humanely euthanized by cervical dislocation after anesthetization by subcutaneous injection of atropine (0.02 mg/kg) when they were unable to reach food or water for more than 24 h and lost 20% body weight.

### Cell Culture

Vero cells and human foreskin fibroblast (HFF) cells were purchased from the ATCC (Manassas, VA, USA) and serially passaged in our laboratories as previously described (Gaskell et al., 2009). The cell lines were cultured in Dulbecco’s modified Eagle’s medium (DMEM, MACGENE, Beijing, China) supplemented with 10% heat-inactivated fetal bovine serum (FBS, Gibco, USA) in a humidified incubator containing 5% CO_2_ at 37°C. The cells were passaged every 3–6 days by trypsinization.

### Parasite Culture and Preparation

The *N. caninum* Nc-1 wild-type strain and the newly constructed NcROP5-deficient strain (described below) were cultured as tachyzoites by serial passages in HFF or vero cells as previously described (Gaskell et al., 2009). Briefly, the parasites were cultured in DMEM (pH 7.4) supplemented with L-glutamine, 2% heat-inactivated FBS, penicillin (100 U/mL) and streptomycin (100 μg/mL) (Sigma–Aldrich, St. Louis, MO, USA) at 37°C in an atmosphere with 5% CO_2_. The parasites were harvested by filtering through a 5.0 μm pore filter (Millipore, MA, USA), centrifuged, washed twice with cold phosphate-buffered saline (PBS), and centrifuged (2,000 rpm for 10 min each time) (Zhang et al., 2007).

### Construction of the NcROP5-Deficient *N. caninum* Strain

The pTCR-CD plasmid was used at the Key Laboratory of Animal Parasitology (Beijing City, China) as previously described (Li et al., 2016). The plasmid contains the chloramphenicol resistance gene (CmR), red fluorescence protein gene (RFP), 5-fluorine cytosine gene (CodA), and ampicillin resistance gene (Amp). *Nctublin* promoter was used to drive the CmR-RFP fusion gene and CodA gene expression. The fragments of the *NcROP5* 5′ (2826 bp) and 3′ UTR regions (2297 bp) were amplified using the primer pairs F1/R1 and F2/R2 (Supplementary Table S1), which were designed based on the *N. caninum* Liverpool protein coding gene sequence^1^ (FR823393, Chromosome: XII). Primers F1, R1, F2, and R2 introduced KpnI, XhoI, XmaI, and SpeI sites, respectively (Supplementary Table S1). After amplification, the fragments were cloned into a pEASY-T-Blunt vector (TransGen Biotech Co., Ltd, Beijing). Then the DNA insert was sequenced (Beijing Sunbiotech Co. Ltd., China) and analyzed by blast in ToxoDB^1^ to confirm the authenticity of the cloned sequence. All alignments were performed using DNAMAN version v5.2.2 and the correct plasmids were double digested with KpnI and XhoI for the 5′ UTR and XmaI and SpeI for the 3′ UTR (NEB, USA). Then, the fragments were ligated to the pTCR-CD vector to produce the *NcROP5* gene deleted vector, pTCR-NcROP5-CD KO.

All constructs were verified by sequencing (Beijing Sunbiotech Co. Ltd, China). After digestion with NotI (NEB, Beijing), the linearized pTCR-NcROP5-CD KO plasmids were purified using ethanol precipitation and then resuspended using cytomix (Hui et al., 2014). Nc-1 tachyzoites (1 × 10^7^), mixed with 50 μg of the above plasmids, were transferred to a 0.4 mm gap cuvette and electroporated with 2 kV at 25 μFd and 50 Ω with the Gene Pulser Xcell electroporation system (Bio-Rad, USA). Then the parasites cultured in an atmosphere containing 5% CO_2_ at 37°C for 24 h prior to the addition of chloramphenicol (20 μM) and 5-fluorine cytosine (40 μM). The parasites were cultured consecutively to the 8th generation and then screened to confirm the purity of the selected strains.

For complementation analysis, pDMG plasmid (Hui et al., 2014; Tao et al., 2014; Li et al., 2016) was used to express NcROP5 in the ΔNcROP5 strain. The complete coding sequence of NcROP5 was amplified using primers F3/R3 (Supplementary Table S1) that appended the flanking EcoRV and AvrII restriction endonuclease sites. The amplification products were introduced into the same sites of a modified pDMG plasmid with GFP replaced by HA (Li et al., 2016). The pDMG-NcROP5-HA vector was electroporated into the ΔNcROP5 strain. The transgenic parasites were grown under pyrimethamine selection pressure. The selected NcROP5 complementary strain, named as iΔNcROP5, was identified by western blotting and immunofluorescence assays (IFAs).

### Validation of the ΔNcROP5 Strain and the Complemented Strain

#### PCR

To screen the *NcROP5* knockout parasites (ΔNcROP5), we designed primers targeting the *NcROP5* fragment (ToxoDB: *NCLIV_060730*, 800 bp) to detect if the gene could be amplified from different clones. Polymerase chain reaction (PCR) was conducted using the T100^TM^ Thermal Cycler (Bio-Rad, USA) with PCR SuperMix (TransGen, China) following manufacturer’s instructions. The primer sequences were F4/R4 (Supplementary Table S1). The *Nc5* gene (336 bp) served as the internal reference with the primer pair F5/R5 (Supplementary Table S1). The PCR conditions were as follows: 95°C for 5 min, 30 cycles of 95°C for 30 s, 56°C for 30 s and 72°C for 1 min, and 72°C for 10 min. The PCR product was identified using electrophoresis.

#### Western Blot

Purified parasites (Nc-1, three ΔNcROP5 clones and one iΔNcROP5) were lysed using RIPA buffer (Beyotime, Beijing) supplemented with a cocktail of protease inhibitors (Sigma, USA). The lysates were resolved on a 10% (w/v) SDS-PAGE gel. After electrophoresis, separated strips were transferred onto polyvinylidene fluoride (PVDF) membranes (Millipore, MA, USA) together with a visible pre-stained protein marker (TransGen Biotech Co., Ltd, Beijing). The membranes were blocked with 5% (w/v) skim milk (BD Difco, USA) in PBS for 1 h at 37°C and then incubated with the mouse anti-rNcROP5 antibody (Tao et al., 2014) (National Animal Protozoa Laboratory in China Agricultural University, 1:500) for ΔNcROP5 and the mouse anti-HA antibody (Sigma–Aldrich, 1:500) for iΔNcROP5. The *N. caninum F*-actin subunit beta (*NcActin*, National Animal Protozoa Laboratory in China Agricultural University) was used as a loading control and was incubated with the rabbit anti-rNcActin antibody (1:2000). After five washes with PBST (1% Tween-20), the membranes were incubated for 1 h with a horseradish peroxidase (HRP)-labeled goat anti-mouse IgG (H+L) secondary antibody (Sigma, USA) diluted 1:5000 and an HRP-labeled goat anti-mouse IgG secondary antibody (Sigma, USA) diluted 1:10,000 in PBS with 5% BSA. Finally, the reactive bands were visualized using enhanced chemiluminescence reagents (CoWin Biotech Co., Ltd, Beijing).

#### Immunofluorescence Assay (IFA)

Immunofluorescence Assay was used to detect the *NcROP5* subcellular localization as previously described (Li et al., 2016). Appropriate numbers of parasites were coated on glass coverslips in 12-well plates and then fixed in 4% paraformaldehyde, permeabilized with 0.25% Triton X-100 and blocked with 3% BSA. Subsequently, the cells were incubated with a mouse anti-HA monoclonal antibody, mouse anti-NcROP5 and rabbit anti-NcSRS2 antibody (National Animal Protozoa Laboratory in China Agricultural University) followed by FITC-conjugated goat-anti mouse IgG (H+L), Cy3-conjugated goat-anti mouse IgG (H+L), and Cy3-conjugated goat-anti rabbit IgG (H+L) (Sigma, USA). The nuclear DNA was stained with Hoechst33258 (Sigma, USA). Extracellular iΔNcROP5 parasites were identified by IFA. The images were obtained using a Leica confocal microscope system (Leica, TCS SP52, Germany).

### Invasion Assays

Cell invasion assays were performed using the following protocol (Tao et al., 2014). Freshly egressed parasites (Nc-1, ΔNcROP5 and iΔNcROP5 strains) were allowed to settle onto the HFF monolayers on 12-well plates for 30 min at 37°C prior to incubation. Extracellular parasites were removed by washing three times with PBS. The cells were incubated for 20 h prior to IFA using anti-NcSRS2 primary and FITC-conjugated secondary antibodies (1:100 dilution). The number of vacuoles representing successful invasion parasites was counted under a fluorescence microscope. A total of three replicates (each clone in duplicate) were performed.

### Plaque Assay

Plaque assays were set up immediately following the initiation of the invasion assays using the same parasite suspensions. Nc-GFP strain (kindly provided by Professor Xuenan Xuan, Obihiro University of Agriculture and Veterinary Medicine, Japan), as a null vector constructing group, which is a transgenic parasite by transfecting Nc-1 wild-type strain with the pDMG plasmid, was propagated as tachyzoites by serial passages in HFF cells as previously described (Gaskell et al., 2009). The plaque assay was performed as previously described (Hui et al., 2014). Five hundred parasites were added to each well of a 6-well plate containing confluent host cell HFF monolayers. The plate was incubated for 7 days in a 37°C incubator with 5% CO_2_, fixed with 4% paraformaldehyde for 15 min and stained with 2% crystal violet for 15 min at room temperature. The stained wells were washed with deionized water, air dried and visualized using a fluorescence microscope (IX71, Olympus, Japan). The plaque area measurement was performed as previously described (Roos et al., 1994).

### Replication Assay

The Nc-1, ΔNcROP5 and iΔNcROP5 parasites were harvested and counted. Five-hundred parasites were added to confiuent vero and HFF cells on 25 mm coverslips. The coverslips were allowed to sit at 37°C for 30 min in DMEM with 20% FBS and then washed three times with PBS. The coverslips were incubated at 37°C for 24 h in DMEM with 2% FBS and then fixed with 4% paraformaldehyde for 15 min. IFAs were performed using anti-NcSRS2 primary (Tao et al., 2014) and FITC-conjugated secondary antibodies (Proteintech, USA). The number of parasites per vacuole was counted under a fluorescence microscope. The experiments were performed in triplicates and repeated three times, and 200 vacuoles were counted per coverslip.

### Egress Assay

*Neospora caninum* tachyzoites, as well as *T. gondii*, must form parasitophorous vacuole (PV) after invading the host cell (Tilley et al., 1997). They proliferate in the PV and then egress out of PV. Then, the tachyzoites invades other cells again (Soldati and Meissner, 2004). In our experiments, parasites were inoculated in 12-well plates containing prepared HFF cells for 30 h. Egress was stimulated with 10 μM of the Ca^2+^ ionophore A23187 from *Streptomyces chartreusensis* (Sigma, USA) for 2 min at 37°C prior to fixation with paraformaldehyde (Williams et al., 2015). The IFA was performed as described above using the mouse anti-NcSRS2 primary and FITC-conjugated secondary molecules. The average number of ruptured vacuoles was determined by counting a minimum of 100 vacuoles per slide and three slides for each experiment. Three independent experiments were performed.

### Gene Expression Assay

Total RNA was extracted from 1 × 10^7^ tachyzoites of the Nc-1 wild-type strain and three ΔNcROP5 clones with TRIzol reagent (Invitrogen, USA). cDNA was synthesized using the EasyScript First-Strand cDNA Synthesis SuperMix kit (TransGen, China). Specific primers were designed using Primer Premier 5.0 (Hui et al., 2014), including primers for rhoptry necks (*RON2* and *RON4*), rhoptrys (*ROP4, ROP5, ROP7, ROP16*, and *ROP17*), dense granules (*GRA2, GRA6*, and *GRA7*) and the endogenous reference gene *NcActin* (Supplementary Table S1). The specificity of these primers was evaluated using conventional quantitative real-time PCR (qRT-PCR). The qRT-PCR was conducted using the ABI Prism 7500 System (Biosystems Inc., USA) with SYBR Green II (Takara Biotechnology, Dalian, Co., Ltd) following manufacturer’s instructions. The resulting RNA concentrations were normalized using Ncactin (Wang et al., 2017), and the relative expression levels of the target genes were analyzed using the ABI Prism 7500 software v2.0.5 (Biosystems Inc., USA). The RT-PCR conditions were as follows: 94°C for 5 s, 40 cycles of 94°C for 5 s and 60°C for 30 s. The relative expression of genes was calculated using the 2^-ΔΔCt^ method and standard deviation was calculated from three replicates (Cárdenas-Mondragón et al., 2016; Gagnaire et al., 2016).

### Label-Free LC-MS/MS Assay

The *N. caninum* Nc-1 and ΔNcROP5 strains were cultured as tachyzoites in HFF cells as previously described (Gaskell et al., 2009) for 96 h. For each parasite, a total of 5 × 10^7^ tachyzoites were harvested and isolated by washing in cold PBS, centrifugation, resuspension in cold PBS, syringing three times through a 27-gaure needle, filtering through a 5.0 μm pore filter (Millipore, MA, USA), washing three times with PBS, and finally centrifugation at 1800 rpm for 10 min (Tao et al., 2014). Then the parasites were ultrasonically lysed in protein lysis buffer (8.0 M urea, 100 mM pH 8.0 Tris-HCl, 1×cocktail) using an ultrasonic liquid processor (Sonic & Materials INC., USA). The lysates were sonicated five times for 2 s each at 30% amplitude. Then the sonicated lysates were centrifuged for 30 min at 4°C at 12000 *g* and obtained the supernatants (Deutsch et al., 2014). After quantitative assay by the Bradford method (Bradford Protein Assay kit) (Deutsch et al., 2014), 60 μg protein was reduced, alkylated and trypsin digested. Each sample was separated by a high performance liquid chromatography (HPLC) system (Ultimate 3000, Thermo Scientific), and analyzed by mass spectrometry (Q-Exactive HF, Thermo Scientific). The spectra were identified and analyzed by the Mascot software (Thermo Scientific, USA) and Proteome discoverer 2.1 (Thermo Scientific, USA) on the basis of database listed in Supplementary Table S2 (Sandberg et al., 2012).

### Mouse Virulence Assay

To compare the virulence of the ΔNcROP5 strain with the parental Nc-1 strain and complemented iΔNcROP5 strain, tachyzoites (3 × 10^6^) were injected i.p. into 6-week-old female BALB/c mice (five mice per strain), which were purchased from the Laboratory Animal Center of Academy of Military Medical Sciences (Beijing, China). Survival was monitored for 30 days. To examine the cerebral load of parasites, tachyzoites were injected i.p. into 6-week-old female BALB/c mice at a dose of 1 × 10^6^ (five mice). The mouse brains were examined for parasites on the 30th day (Pan et al., 2014).

### Statistical Analysis

The statistical analysis of all of the data was performed using GraphPad Prism 5 v. 5.01 (San Diego, CA, USA). The results were expressed as mean ± SD and evaluated by non-parametric tests. Values of *P* < 0.05 and *P* < 0.01 were considered statistically significant and very significant, respectively.

## Results

### Successful Construction of the *NcROP5* Knockout Strain

**FIGURE 1:**
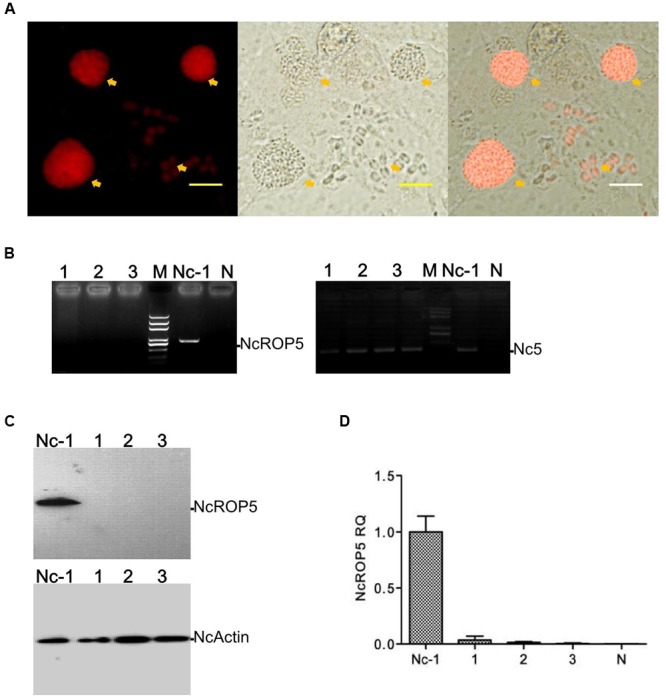
**Identification of the ΔNcROP5 strains. (A)** The *NcROP5*-disrupted (ΔNcROP5) strain was identified using a fluorescent inverted microscope. The positive parasites showed red fluorescent (Yellow arrow). **(B)** ORF-specific PCR confirming the deletion of the *NcROP5* gene locus. An expected 800 bp band of the ROP5 gene from the parental Nc-1 strain was amplified compared to the absence of this band in the three ΔNcROP5 clones (1, 2, and 3) and in the plasmid DNA (negative control, N). *Nc5* gene served as a *Neospora caninum* specific gene. **(C)** Western blots with *NcROP5* anti-mouse antibodies with *NcROP5* of 61 kDa in the parental Nc-1 strain. No NcROP5 protein was detected in the ΔNcROP5 monoclonal strains (1, 2, and 3). *N. caninum F-*actin subunit beta (*NcActin*) was used as a loading control. **(D)** Quantitative RT-PCR was used to analyze the transcription levels of the *NcROP5* gene in the ΔNcROP5 clones (1, 2, and 3) and compared with the parental strain (Nc-1). Each bar indicates the relative quantity (RQ) ±SD. Scale bar, 10 μm.

Using the knockout plasmid, pTCR-CD, we successfully constructed the *NcROP5* knockout strain. To use the plasmid for deleting a gene, we ligated the 5′ UTR and 3′ UTR regions of the target gene. Then, we analyzed the genome of *N. caninum Liverpool* strain in ToxoDB^2^ and identified the sequences with high identity to *TgROP5*, which were *NcLIV_060730, NcLIV_060740*, and *NcLIV_060741*. Compared to the tandem cluster of polymorphic alleles of *TgROP5* (*TgROP5A, TgROP5B*, and *TgROP5C*) (Behnke et al., 2011), we believed that *NcROP5* would contain *NcROP5A, NcROP5B*, and *NcROP5C*, coded by the above three genes, respectively. Homology of nucleotide and protein sequence between *NcLIV_060730* and *TgROP5A* were 52.72 and 76.76%, respectively. Since *TgROP5* is an important virulence factor of *T. gondii* (Behnke et al., 2012), we suspected that NcROP5 might play an active role in *N. caninum* virulence. To examine the physiological role of ROP5 in the Nc-1 strain, *NcROP5* KO parasites were generated by targeted gene disruption. The non-coding region (UTR) sequences of *NcROP5, NcROP5* 5′ UTR and 3′ UTR, were cloned (**Supplementary Figure S1A**) and ligated to the pTCR-CD vector, forming pTCR-NcROP5-CD KO (**Supplementary Figure S1B**). The plasmid was identified by restriction enzyme digestion (**Supplementary Figure S1A**) and sequenced and then transfected into Nc-1. Theoretically, the entire ROP5 coding region, containing *NcROP5A, NcROP5B*, and *NcROP5C*, could be replaced with the CmR and RFP genes (**Supplementary Figure S1C**). RFP was expressed at normal levels in the suspected *ROP5* mutants (**Figure 1A**). Following CmR selection of the RFP and 5-fluorine cytosine-negative parasites, we isolated three mutants out of 218 clones.

To generate the *ROP5* gene-deficient Nc-1 strain, the *NcROP5* gene was targeted and replaced by CmR-RFP. PCR was used to confirm the deletion of the *NcROP5* gene. *NcROP5* gene amplification was negative in the parasite genomes (**Figure 1B**). NcROP5 deficiency was confirmed using western blotting (**Figure 1C**) and RT-PCR (**Figure 1D**). We also successfully generated a strain with complete knockout of the *NcROP5* gene. These results suggested that *NcROP5* was not an essential, surviving gene in the parasites.

### Successful Construction of the *NcROP5* Complementary Strain

**FIGURE 2:**
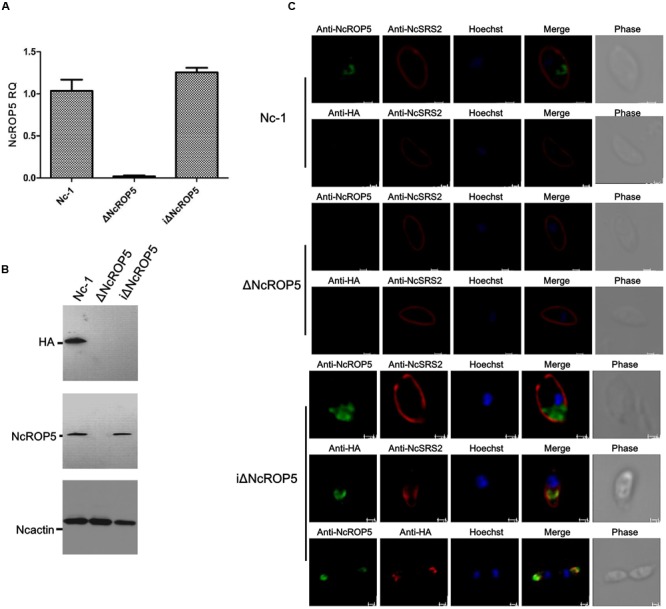
**Identification of ΔNcROP5 complementary strain, iΔNcROP5. (A)** RT-PCR analysis of *NcROP5* gene transcription. **(B)** Western blotting analysis of Nc-1, ΔNcROP5 and iΔNcROP5 tachyzoites. The expected band was detected by the anti-HA monoclonal antibody and anti-rNcROP5 polyclonal antibody. NcActin was used as the control. **(C)** IFA analysis of iΔNcROP5 tachyzoites. The HA-tagged ROP5 and ROP5 proteins (green) were localized at the anterior end of the parasites, and HA co-localized with ROP5. The shapes of parasites were stained with anti-NcSRS2 (red), and the nuclear DNA was stained with Hoechst (blue). Scale bar, 1.0 μm.

To verify the biological role of *NcROP5*, we obtained the complete coding sequence of *NcROP5A* without the termination codon TAA (NcLIV_060730, 1647 bp, **Supplementary Figure S2A**) and generated the complementary plasmid pDMG-NcROP5-HA. The expression of *NcROP5* with an HA-tag and stop codon TAA at the C-terminus was driven under the control of the NcGRA1 promoter (**Supplementary Figure S2B**). We correctly identified the plasmid by restriction enzyme digestion and DNA sequencing (**Supplementary Figure S2A**), and then electroporated it into one of the ΔNcROP5 strains. After repeated passage, we selected strains that stably expressed *NcROP5* (named as iΔNcROP5). *NcROP5* expression in the complementary strain based on WB and RT-PCR was comparable to the expression of the wild strain (**Figures 2A,B**). IFA was used to analyze the subcellular localization of *NcROP5* in the iΔNcROP5 strains using a mouse anti-HA monoclonal antibody. *NcROP5* was co-localized with HA at the anterior end of the extracellular parasites (**Figure 2C**).

### The *NcROP5* Gene Is Critical in the Invasion and Proliferation of *N. caninum*

**FIGURE 3:**
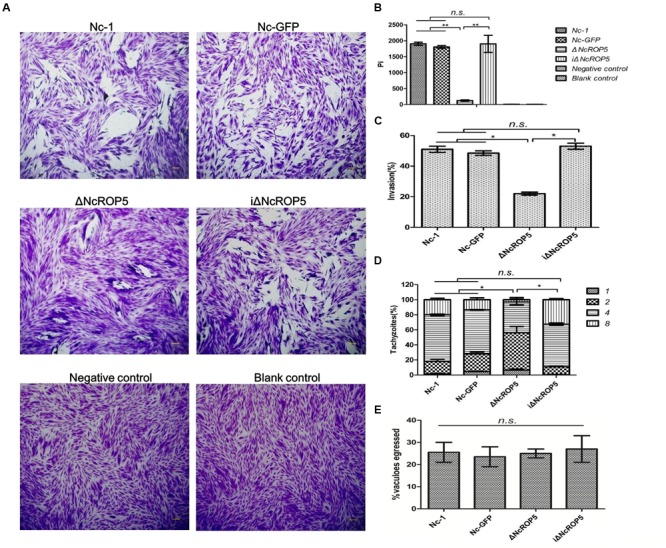
**Deletion of the *NcROP5* gene affects the Nc-1 strain phenotype. (A,B)** Plaque assays showed very low plaque formation of the ΔNcROP5 strain compared with the strains harboring *NcROP5*. The assay was performed three times independently, and the plaque area was quantified from three independent experiments. At least 10 fields were quantified per strain in every experiment. Each bar indicates mean ± SD; “Pi” indicates pixels of each plaque. **(C)** The ΔNcROP5 strain possess significantly reduced cell invasion compared with the Nc-1, Nc-GFP, and iΔNcROP5 strains. The number of parasite vacuoles was counted after immunofluorescent staining with anti-NcSRS2 antibodies in the cell invasion assay. **(D)** Intracellular replication assay showed that ΔNcROP5 strain proliferated more slowly than the other three strains. The IFA was performed with anti-NcSRS2 antibodies. At least 100 vacuoles were examined for each per strain. **(E)** Egress assay showed no difference between strains indicating that *NcROP5* is not involved in parasite egress. ^∗^*P* < 0.01 and ^∗∗^*P* < 0.001 (Non-parametric tests). Scale bar, 10 μm.

Plaque formation measures the viability of parasites in cell culture and reflects parasite motility on the surface of the host cell layer, invasion, intracellular growth and egress (Plattner et al., 2008). We assessed the ΔNcROP5 strain using the plaque assay. The ΔNcROP5 strain formed noticeably smaller and considerably fewer plaques than the parental strain and the complementary strain (**Figures 3A,B**). The ΔNcROP5 strain showed significant weakening of host cell invasion (**Figure 3C**). Intracellular replication assays were performed to investigate if deleting *ROP5* in the Nc-1 strain weakened the ability of the parasite to proliferate in host cells. Parasites that invaded host cells 24 h after inoculation were analyzed for intracellular growth by counting the number of parasites per vacuole. Due to the lack of synchronization of host cell invasion, the intracellular vacuoles contained 1, 2, 4, or 8 parasites. However, intracellular replication of ΔNcROP5 in Vero and HFF cells was lower than that of the wild type parasite (Nc-1). As shown in **Figure 3D**, the distribution of the eight parasites per vacuole was significantly lower for the ROP5 knockout strain than that for Nc-1, Nc-GFP, and iΔNcROP5, indicating that the intracellular replication of the ΔNcROP5 strain was slower than that of the other three strains. However, there was no significant difference in egress between the four strains (**Figure 3E**). These findings indicated that *NcROP5* expression in *N. caninum* is a critical determinant of parasite phenotype, including host cell invasion and proliferation.

### Identification of Peptides and Proteins in Nc-1 and ΔNcROP5

To analyze the cause of the ΔNcROP5 phenotype changes, we compared protein expression levels in Nc-1 and ΔNcROP5. Nc-1 and ΔNcROP5 tachyzoites were analyzed by label-free LC-MS/MS for quantification of peptides. A total of 18,809 peptides with FDR ≤ 0.01 were identified, and a total of 1149 proteins with expression difference ≥ 1.2-fold between the two strains were functionally annotated according to the *N. caninum* genome annotation in the ToxoDB database (**Supplementary Figure S3A** and Table S3). The three main annotation types were biological process, cellular component and molecular function (**Supplementary Figures S3B–D**). The differentially expressed proteins were classified into 31 functional groups (**Supplementary Figure S3E**), with biological process possessing 12 GO (gene ontology annotation) terms, cellular component 10 GO terms, and molecular function 9 GO terms. Cellular process and metabolic process were major biological processes (32 and 31%, respectively), cell part and cell are major cellular components (25% each), and binding and catalytic activities were major molecular functions (53 and 37%, respectively).

### Molecularly Enriched Pathways Associated with Differentially Expressed Proteins in Nc-1 and ΔNcROP5

To further identify biological pathways of the differentially expressed proteins, the number of proteins expressed in Nc-1 and ΔNcROP5 and differences between the two strains are shown in **Supplementary Figure S4A**. Demographic analysis demonstrated that 76 proteins were increased and 89 proteins were decreased with a 2.0-fold change; 198 proteins were increased and 252 proteins were decreased with a 1.5-fold change; and 477 proteins were increased and 671 proteins were decreased with a 1.2-fold change. We predicted that 477 increased proteins participated in 92 biological pathways and 671 decreased proteins participated in 88 biological pathways by KEGG pathway analysis^3^. The differentially expressed proteins were matched with the proteins annotated in the KEGG pathway database, and they were compared to determine their involvement in the KEGG pathway. We found that five KEGG pathways were clearly affected (*P* ≤ 0.05) (**Supplementary Figure S4B** and Table S4), followed by mismatch repair, SNARE interactions in vesicular transport, glycosylphosphatidylinositol (GPI)-anchor biosynthesis, ABC transporters and RNA degradation.

To better comprehend the causes of phenotypic differences, we analyzed representative differentially expressed proteins and uncommented proteins by gene and sequence alignment. Blast analysis (ToxoDB-28_*Neospora caninum*_Annotated Proteins. fasta) showed that increased proteins included apetela 2 (AP2) family proteins (*NCLIV_063920, NCLIV_032930*, and *NCLIV_059950*) and dense granule protein (*NCLIV_004260*), and decreased proteins included cell division and proliferation proteins (*NCLIV_004280, NCLIV_005020, NCLIV_069400, NCLIV_065020, NCLIV_067140*, and *NCLIV_009310)*, and secreted proteins (*NCLIV_038100*) (**Table 1**).

**Table 1:** Changed proteins in the ΔNcROP5 group.

ToxoDB accession number	Product description	Fold change
NCLIV_063920	AP2 domain transcription factor AP2XII-4	13.69↑
NCLIV_032930	AP2 domain transcription factor AP2VIII-2	6.21↑
NCLIV_059950	AP2 domain transcription factor AP2XI-5	2.32↑
NCLIV_004280	Cell division protein	3.31↓
NCLIV_005020	Putative PUA domain-containing, cell cycle regulator protein	2.82↓
NCLIV_069400	VEG-inner membrane protein	2.68↓
NCLIV_038100	Microneme protein 5(MIC5)	2.64↓
NCLIV_065020	Putative actin-like protein 3b	2.45↓
NCLIV_067140	Myosin, related	2.36↓
NCLIV_009310	Putative trafficking protein particle complex subunit 3	2.12↓

*(↑) Increased; (↓) Decreased.*

**FIGURE 4:**
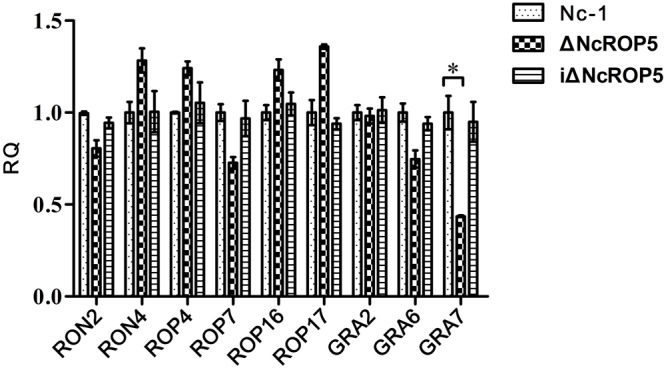
**The transcription of differential genes in Nc-1, ΔNcROP5 and iΔNcROP5.** Quantitative RT-PCR analyses of the transcription levels of the indicated genes in ΔNcROP5 parasites compared with the parental strain (Nc-1) and the complementary strain (iΔNcROP5). Each bar indicates the relative quantity (RQ) ±SD. The RQs of the ΔNcROP5 and iΔNcROP5 parasites were expressed as fold of the expression by Nc-1, which was artificially set as 1. The data presented are representative of three independent experiments each performed in duplicate. ^∗^*P* < 0.05.

Rhoptry neck proteins (RONs), ROPs and dense granule proteins (GRAs) were related to invasion, proliferation, and formation of PV (Sinai and Joiner, 2001; Dubremetz and Lebrun, 2012; Nolan et al., 2015). To detect whether these genes were affected by *NcROP5*, we selected *RON2, RON4, ROP4, ROP7, ROP16, ROP17, GRA2, GRA6*, and *GRA7* genes to evaluate their mRNA expression. Quantitative RT-PCR (qRT-PCR) analysis of 1 × 10^7^ Nc-1, ΔNcROP5 and iΔNcROP5 tachyzoites was conducted to determine the potential involvement of *NcROP5* in transcriptional regulation of other related genes. The qRT-PCR analysis indicated that *RON2* (*R*^2^ = 0.9838, slope = -3.392), *RON4* (*R*^2^ = 0.9849, slope = -3.425), *ROP4* (*R*^2^ = 0.9961, slope = -3.381), *ROP7* (*R*^2^ = 0.9918, slope = -3.466), *ROP16* (*R*^2^ = 0.9907, slope = -3.372), *ROP17* (*R*^2^ = 0.9899, slope = -3.373), *GRA2* (*R*^2^ = 0.9911, slope = -3.407), and *GRA6* (*R*^2^ = 0.9951, slope = -3.475) were not altered at the transcription level in ΔNcROP5. Notably, *NcGRA7* (*R*^2^ = 0.9879, slope = -3.331) expression was significantly down-regulated by approximately twofold in ΔNcROP5 (**Figure 4**). All genes have similar amplification efficiency to the reference gene *Ncactin* (*R*^2^ = 0.9912, slope = -3.392). The results suggested that *NcROP5* expression might affect the expression of other genes, including *NcGRA7*.

### Knockout of *NcROP5* Reduced Parasite Virulence

**FIGURE 5:**
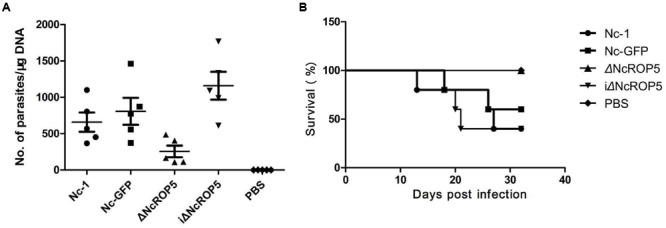
**The ROP5 gene is critical in *N. caninum* virulence. (A)** Brain parasite load of ΔNcROP5 was significantly reduced compared to Nc-1, Nc-GFP, and iΔNcROP5. BALB/c mice (*n* = 5) were injected i.p. with 1 × 10^6^ tachyzoites and the number of parasites in the brain was determined 30 days after parasite inoculation. **(B)** All ΔNcROP5-inoculated mice survived compared with 40–60% survival of Nc-1, Nc-GFP and iΔNcROP5-inoculated animals. BALB/c mice (*n* = 5) were infected with 3 × 10^6^ tachyzoites and monitored for 30 days. Two independent experiments were performed.

To investigate if *NcROP5* is necessary for Nc-1 virulence, mice were infected intraperitoneally with 1 × 10^6^ Nc-1, Nc-GFP, ΔNcROP5, or iΔNcROP5 tachyzoites, followed by observation of parasite load in the brain and survival rate. Significant differences were observed between the ΔNcROP5 group and the other groups (Nc-1, Nc-GFP and iΔNcROP5). Signs of illness such as rough coats, inactivity, or nervous system signs (hind limb weakness, head tilting, or walking in circles) were observed 2 or 5 days before death. In addition, rough hair coats and reduced activity were observed in 2 Nc-1-infected mice from day 28 post-infection, 1 Nc-GFP-infected mouse from day 29 post-infection and 1 iΔNcROP5-infected animal from day 26 post-infection. There were no clinical signs in ΔNcROP5-infected mice during the 30 days observation period. The cerebral parasite load of the ΔNcROP5 group was approximately one-fourth of the load in the Nc-1 and Nc-GFP groups. The iΔNcROP5 group had the most parasites in the brain (**Figure 5A**). Consistent with the low parasite load in the brain, the survival rate of the ΔNcROP5-infected animals was higher than that of the other groups. Actually, all ΔNcROP5-infected mice survived, compared with the survival rate of 40–60% for the other groups (**Figure 5B**). These results demonstrate a requirement of *NcROP5* for Nc-1 virulence.

## Discussion

*Neospora caninum* and *T. gondii* are obligate intracellular parasites that belong to the phylum *Apicomplexa*, which includes *Cryptosporidium, Eimeria, Plasmodium*, and *Theileria* (Hajj et al., 2007; Plattner et al., 2008). *Toxoplasma* is a widely used model organism for studying protein functions in *Apicomplexa* (Shen et al., 2014). In *T. gondii*, the methods of gene deletion and protein expression regulation have been developed to study protein functions and provided valuable information (Payne et al., 2011). However, related research reports about functions of proteins in *N. caninum* are limited to epidemiological studies, the expression and localization of cloned genes, and immunogenicity (Liu et al., 2013; Li et al., 2014; Hecker et al., 2015; Pastor-Fernández et al., 2016). The overexpression of some genes and addition of labeling with biotin in *N. caninum* have been reported (Tao et al., 2014; Mota et al., 2016). Few reports on *N. caninum* have investigated the loss of one or more genes and their effects on gene functions (Mota et al., 2016).

The genome-editing technique is very important and has advanced researches on the roles of gene functions in some organisms. We constructed a pTCR-CD knockout plasmid and used the plasmid to edit genes in *N. caninum* successfully with the example of the *NcROP5* gene. This step was a very positive development for research on *N. caninum* protein functions. The roles of *NcROP5* were partially deduced using the strain lacking the *NcROP5* gene based on the PCR assay results, gene transcription levels and protein expression levels. The study provides a reference method and lays the foundation for further studies on NcROP5 functions and other tachyzoite proteins. We selected three ΔNcROP5 monoclonal strains and observed their phenotypes. The phenotypes of the ΔNcROP5 strains were similar, with a weakened invasion capacity, slower intracellular proliferation rate and smaller sized plaques. However, there were significant differences compared to the Nc-1, Nc-GFP, and ΔNcROP5 complement strains. The slight phenotypic differences between iΔNcROP5 and the reference strains might be due to the efficient promoter NcGRA1 of pDMG-NcROP5-HA, while ROP5 is encoded in a multicopy gene in the reference strain Nc-1. The results indicated that the *NcROP5* gene played an important role in *N. caninum* virulence. The phenotypic changes in the ΔNcROP5 strains were probably caused by the abnormal expression of other genes associated with *NcROP5*.

Proteomics analysis was performed to explore the proteins expression characteristics of Nc-1 and ΔNcROP5, but regrettably there were few interesting proteins secreted by microneme, rhoptry, or dense granule. GRA7 transcription level was reduced in ΔNcROP5 parasites, and for the reason that qRT-PCR was more sensitive than label-free analysis. In this part, we noticed some mysterious proteins, AP2 protein family. The apicomplexan AP2 (ApiAP2) family of binding proteins might be a major class of transcriptional regulators in *Apicomplexan* (Radke et al., 2013). There are 68 *ApiAP2*-encoding genes predicted in the *Toxoplasma* genome (Altschul et al., 2010). We crudely inferred the functions of the genes according to other *Apicomplexan* parasites, because they have not been fully studied in *N. caninum*. In *T. gondii, AP2XI-5* targets more than 300 gene promoters and actively controls the transcription of these genes. *AP2XI-5* was reported to be enriched at numerous active promoters, especially crucial virulence factors such as the rhoptry and microneme proteins (Walker et al., 2013b). In addition, *TgAP2XI-4* regulates bradyzoite gene expression during parasite differentiation and cyst formation (Walker et al., 2013a), and *TgAP2XI-3* dampens proliferation of parasites (Lai et al., 2012). In our study, the expression levels of *AP2VIII-2, AP2XII-4*, and *AP2XII-5* were increased and *AP2VIII-3* was not detected in the ΔNcROP5 group. These findings might be related to the loss of *NcROP5* or abnormal expression of virulence factors NcROPs, MICs, GRAs and some metabolic enzymes as result of *NcROP5* deletion. Meanwhile expression of some proteins, such as *actin, myosin*, and cell division protein, was decreased in the ΔNcROP5 strain, consistent with the slow proliferation of ΔNcROP5. The lower expression of *microneme protein 5* (*MIC5*) was one possible reason for the weaker invasion capacity of ΔNcROP5, because MIC5 as a secreted protein modulates parasite adhesion and invasion (Brydges et al., 2008; Gaji et al., 2011).

*Toxoplasmosis gondii* strains are primarily divided into three types based on genomic analysis (type I, type II, and type III) (Grigg et al., 2001; Su et al., 2003). Previous studies identified a closely related *ROP5* gene of *T. gondii* that was absolutely critical for pathogenesis in mice; the deletion of this gene resulted in a complete loss of virulence in mice even following infection with 10^6^ parasites (Reese and Boothroyd, 2011). For *N. caninum*, various authors have described evidence of different toxicity among isolates. They demonstrated Nc-liv and Nc-Spain 7 had a higher virulence than Nc-1 (Regidorcerrillo et al., 2010; Caspe et al., 2012), and Nc-Spain 2H, Nc-Spain 3H, Nc-Spain 5H, Nc-Spain 6, Nc-Spain 7, and Nc-Spain 9 isolates showed different parasite burdens and brain lesions during the late phase of infection (Pereira et al., 2010). Although among *N. caninum* isolates, Nc-1 is less virulent in cattle than other isolates, it still leads to nervous system deficiency and deaths (Pan et al., 2014). In our study, the pathogenicity of the ΔNcROP5 strain from Nc-1 was greatly decreased, similar to *TgROP5*. The phylogenetic tree was showed that *NcROP5* is closely related to *TgROP5* (Ghorbal et al., 2014). *ROP5* likely has similar functions in *Apicomplexa* and mediates parasite virulence (Reese and Boothroyd, 2011; Walzer et al., 2013). In *Toxoplasma, ROP5, ROP18*, and *GRA7* form a complex that plays critical roles in parasite virulence (Hermanns et al., 2016). *ROP5* and *ROP17* were also reported to control *T. gondii* RH strain virulence through the complex (Etheridge et al., 2014). However, *ROP18* has been proven to be a pseudogene and not a virulence factor in *N. caninum* (Reid et al., 2012). Surprisingly, the loss of *NcROP5* led to a reduction in *NcGRA7* transcription in our study. In contrast, *TgGRA7* bound the host cell immunity-related GTPase A6 and enhanced its polymerization, rapid turnover, and eventual disassembly (Alaganan et al., 2014). *TgGRA7* was considered a virulence factor, which was in contrast to the *NcGRA7* immunogenicity analysis (Vemulapalli et al., 2007; Alaganan et al., 2014). Therefore, further research is needed to clarify the association between *NcROP5, NcROP17*, and *NcGRA7*, although we have defined *NcROP5* as a factor that affects *N. caninum* virulence in mice.

## Conclusion

Our findings here highlight the role of *ROP5* as an important virulence factor in *N. caninum* and may contribute to a better understanding of the possible mechanisms underlying the interactions between parasites and their hosts.

## Author Contributions

Conceived and designed the experiments: LM, JL, and QL. Performed the experiments: LM, ML, and YF. Analyzed the data: LM, XZ, and QL. Contributed reagents/materials/analysis tools: QL and JL. Wrote the paper: LM and QL.

## Conflict of Interest Statement

The authors declare that the research was conducted in the absence of any commercial or financial relationships that could be construed as a potential conflict of interest.

## Footnotes

1 http://ToxoDB.org/toxo/

2 http://toxodb.org/toxo/

3 http://www.genome.jp/kegg

## Supplementary Material

FIGURE S1 **Construction of pTCR-NcROP5-CD KO. (A)** Products of *NcROP5* 5′ UTR and 3′ UTR by PCR and identification of pTCR-NcROP5-CD KO by enzyme digestion. 1: *NcROP5* 5′ UTR; 2: *NcROP5* 3′ UTR; 3, 5: The knockout plasmid pTCR-NcROP5-CD KO; 4: Double enzyme restriction of pTCR-NcROP5-CD KO for *NcROP5* 5′ UTR; 6: Double enzyme restriction of pTCR-NcROP5-CD KO for *NcROP5* 3′ UTR; M1: Trans2K plus DNA marker; M2: Trans15k DNA marker. **(B,C)** Schemes of the targeted disruption of the *NcROP5* gene by double homologous recombination. The pTCR-NcROP5-CD KO plasmid contains the 5′ UTR and 3′ UTR sequences flanking the CmR and RFP marker, which confers resistance to chloramphenicol.

FIGURE S2 **Construction of ΔNcROP5 complementary strain, iΔNcROP5. (A)** Products of *NcROP5A* by PCR and identification of the pDMG-NcROP5-HA plasmid by enzyme digestion. 1: *NcROP5A* coding gene. 2: Double enzyme restricted pDMG-NcROP5-HA. M1: DNA 2000 plus M2: Trans 15K. **(B)** Diagram of the pDMG-NcROP5-HA construct.

FIGURE S3 **Gene ontology analyses of differential proteins in Nc-1 and ΔNcROP5. (A)** Differential analysis between Nc-1 and ΔNcROP5. **(B)** Biological process. Cellular process (31.88%), Metabolic process (31.38%), Single-Organism Process (16.86%), Localization (7.80%), Biological regulation (4.36%), Cellular component organization or biogenesis (3.27%), Response to stimulus (2.68%), Signaling (1.26%), Multicellular organismal process (0.25%), Multi-Organism process (0.08%), Detoxofication (0.08%), Developmental process (0.08%); **(C)** Cellular component. Cell part (24.92%), Cell (24.92%), Organelle (13.85%), Membrane (11.73%), Macromolecular complex (11.62%), Membrane part (6.15%), Organelle part (5.25%), Membrane-enclosed lumen (1.01%), Extracellular region (0.45%), Supramolecular fiber (0.11%); **(D)** Molecular function. Binding (52.84%), Catalytic activity (37.34%), Structural molecule activity (3.67%), Transporter activity (2.66%), Nucleic acid binding transcription factor activity (1.10%), Molecular function regulator (0.92%), Electron carrier activity (0.92%), Antioxidant activity (0.46%), Metallochaperone activity (0.09%); **(E)** Number of proteins involved in biological process, cellular component and molecular function.

FIGURE S4 **The number of differential proteins and classification of enriched pathways in Nc-1 and ΔNcROP5. (A)** Increased and decreased proteins are divided into three groups: differentially expressed proteins with a 1.2-, 1.5-, and 2.0-fold difference. **(B)** Proteins with 1.5-fold difference are categorized by enrichment analysis to 39 pathways.

## References

[B1] Alaganan A. Fentress S. J. Tang K. Wang Q. Sibley L. D. (2014). *Toxoplasma* GRA7 effector increases turnover of immunity-related GTPases and contributes to acute virulence in the mouse. *Proc. Natl. Acad. Sci. U.S.A.* 111 1126–1131. 10.1073/pnas.1313501111 24390541 PMC3903209

[B2] Altschul S. F. Wootton J. C. Zaslavsky E. Yu Y.-K. (2010). The construction and use of log-odds substitution scores for multiple sequence alignment. *PLoS Comput. Biol.* 6:e1000852 10.1371/journal.pcbi.1000852 PMC2904766 20657661

[B3] Arranz-Solís D. Aguado-Martíne A. Müller J. Regidor-Cerrillo J. Ortega-Mora L. M. Hemphill A. (2015). Dose-dependent effects of experimental infection with the virulent *Neospora caninum* Nc-Spain7 isolate in a pregnant mouse model. *Vet. Parasitol.* 211 133–140. 10.1016/j.vetpar.2015.05.021 26104964

[B4] Behnke M. S. Fentress S. J. Mashayekhi M. Li L. X. Taylor G. A. Sibley L. D. (2012). The polymorphic pseudokinase ROP5 controls virulence in *Toxoplasma gondii* by regulating the active kinase ROP18. *PLoS Pathog.* 8:e1002992 10.1371/journal.ppat.1002992 PMC3493473 23144612

[B5] Behnke M. S. Khan A. Lauron E. J. Jimah J. R. Wang Q. Tolia N. H. (2015). Rhoptry proteins ROP5 and ROP18 are major murine virulence factors in genetically divergent south american strains of *Toxoplasma gondii*. *PLoS Genet.* 11:e1005434 10.1371/journal.pgen.1005434 PMC4546408 26291965

[B6] Behnke M. S. Khan A. Wootton J. C. Dubey J. P. Tang K. Sibley L. D. (2011). Virulence differences in Toxoplasma mediated by amplification of a family of polymorphic pseudokinases. *Proc. Natl. Acad. Sci. U.S.A.* 108 9631–9636. 10.1073/pnas.1015338108 21586633 PMC3111276

[B7] Brydges S. D. Harper J. M. Parussini F. Coppens I. Carruthers V. B. (2008). A transient forward-targeting element for microneme-regulated secretion in *Toxoplasma gondii*. *Biol. Cell* 100 253–264. 10.1042/BC20070076 17995454 PMC2663041

[B8] Cárdenas-Mondragón M. G. Ares M. A. Panunzi L. G. Pacheco S. Camorlinga-Ponce M. Girón J. A. (2016). Transcriptional profiling of Type II toxin–antitoxin genes of *Helicobacter pylori* under different environmental conditions: identification of HP0967–HP0968 system. *Front. Microbiol.* 7:1872 10.3389/fmicb.2016.01872 PMC5118875 27920769

[B9] Caspe S. G. Moore D. P. Leunda M. R. Cano D. B. Lischinsky L. Regidor-Cerrillo J. (2012). The *Neospora caninum*–spain 7 isolate induces placental damage, fetal death and abortion in cattle when inoculated in early gestation. *Vet. Parasitol.* 189 171–181. 10.1016/j.vetpar.2012.04.034 22621962

[B10] Deutsch D. R. Fröhlich T. Otte K. A. Beck A. Habermann F. A. Wolf E. (2014). Stage-specific proteome signatures in early bovine embryo development. *J. Proteome Res.* 13 4363–4376. 10.1021/pr500550t 25102770

[B11] Dubey J. P. (1999). Recent advances in *Neospora* and neosporosis. *Vet. Parasitol.* 84 349–367. 10.1016/S0304-4017(99)00044-8 10456423

[B12] Dubey J. P. Schares G. Ortega-Mora L. M. (2007). Epidemiology and control of Neosporosis and *Neospora caninum*. *Clin. Microbiol. Rev.* 20 323–367. 10.1128/CMR.00031-06 17428888 PMC1865591

[B13] Dubremetz J. F. Lebrun M. (2012). Virulence factors of *Toxoplasma gondii*. *Microbes Infect.* 14 1403–1410. 10.1016/j.micinf.2012.09.005 23006855

[B14] Etheridge R. Alaganan A. Tang K. Lou H. J. Turk B. Sibley L. D. (2014). The Toxoplasma pseudokinase ROP5 forms complexes with ROP18 and ROP17 kinases that synergize to control acute virulence in mice. *Cell Host Microbe* 15 537–550. 10.1016/j.chom.2014.04.002 24832449 PMC4086214

[B15] Gagnaire A. Gorvel L. Papadopoulos A. Von Bargen K. Mège J. Gorvel J. (2016). COX-2 inhibition reduces *Brucella* bacterial burden in draining lymph nodes. *Front. Microbiol.* 7:1987 10.3389/fmicb.2016.01987 PMC5149544 28018318

[B16] Gaji R. Y. Flammer H. P. Carruthers V. B. (2011). Forward targeting of *Toxoplasma gondii* proproteins to the micronemes involves conserved aliphatic amino acids. *Traffic* 7 840–853. 10.1111/j.1600-0854.2011.01192.x PMC3115430 21438967

[B17] Gaskell E. A. Judith E. S. John W. P. Dave R. W. Glenn A. M. (2009). A unique dual activity amino acid hydroxylase in *Toxoplasma gondii*. *PLoS ONE* 4:e4801 10.1371/journal.pone.0004801 PMC2653193 19277211

[B18] Ghorbal M. Gorman M. Macpherson C. R. Martins R. M. Scherf A. Lopezrubio J. J. (2014). Genome editing in the human malaria parasite *Plasmodium falciparum* using the CRISPR-Cas9 system. *Nat. Biotechnol.* 32 819–821. 10.1038/nbt.2925 24880488

[B19] Grigg M. E. Bonnefoy S. Hehl A. B. Suzuki Y. Boothroyd J. C. (2001). Success and virulence in Toxoplasma as the result of sexual recombination between two distinct ancestries. *Science* 294 161–165. 10.1126/science.1061888 11588262

[B20] Hajj H. E. Maryse L. Stefan T. A. Henri V. Gilles L. Jean F. O. D. (2007). ROP18 Is a rhoptry kinase controlling the intracellular proliferation of *Toxoplasma gondii*. *PLoS Pathog.* 3:e14 10.1371/journal.ppat.0030014 PMC1797617 17305424

[B21] Hall C. A. Reichel M. P. Ellis J. T. (2005). *Neospora* abortions in dairy cattle: diagnosis, mode of transmission and control. *Vet. Parasitol.* 128 231–241. 10.1016/j.vetpar.2004.12.012 15740860

[B22] Hammoudi P. Jacot D. Mueller C. Di Cristina M. Dogga S. K. Marq J. (2015). Fundamental roles of the golgi-associated toxoplasma aspartyl protease, ASP5, at the host-parasite interface. *PLoS Pathog.* 11:e1005211 10.1371/journal.ppat.1005211 PMC4608785 26473595

[B23] Hecker Y. P. Cantón G. Regidor-Cerrillo J. Chianini F. Morrell E. Lischinsky L. (2015). Cell mediated immune responses in the placenta following challenge of vaccinated pregnant heifers with *Neospora caninum*. *Vet. Parasitol.* 214 247–254. 10.1016/j.vetpar.2015.10.015 26553499

[B24] Hermanns T. Müller U. B. Könen-Waisman S. Howard J. C. Steinfeldt T. (2016). The *Toxoplasma gondii* rhoptry protein ROP18 is an Irga6-specific kinase and regulated by the dense granule protein GRA7. *Cell. Microbiol.* 18 244–259. 10.1111/cmi.12499 26247512 PMC5061101

[B25] Hui W. Tao L. Jing L. Muzi L. Huizhu N. Qun L. (2014). A nuclear factor of high mobility group box protein in *Toxoplasma gondii*. *PLoS ONE* 9:e111993 10.1371/journal.pone.0111993 PMC4219823 25369210

[B26] Lai B. S. Witola W. H. El Bissati K. Zhou Y. Mui E. Fomovska A. (2012). Molecular target validation, antimicrobial delivery, and potential treatment of *Toxoplasma gondii* infections. *Proc. Natl. Acad. Sci. U.S.A.* 109 14182–14187. 10.1073/pnas.1208775109 22891343 PMC3435209

[B27] Li J. He P. Yu Y. Du L. Gong P. Zhang G. (2014). Detection of *Neospora caninum*-DNA in feces collected from dogs in Shenyang (China) and ITS1 phylogenetic analysis. *Vet. Parasitol.* 205 361–364. 10.1016/j.vetpar.2014.06.036 25047704

[B28] Li M. Wang H. Liu J. Hao P. Ma L. Liu Q. (2016). The apoptotic role of metacaspase in *Toxoplasma gondii*. *Front. Microbiol.* 6:1560 10.3389/fmicb.2015.01560 PMC4717298 26834715

[B29] Liu G. Cui X. Hao P. Yang D. Liu J. Liu Q. (2013). GRA 14, a novel dense granule protein from *Neospora caninum*. *Acta Biochim. Biophys. Sin.* 45 607–609. 10.1093/abbs/gmt036 23722878

[B30] Lyon C. (2010). Update on the diagnosis and management of *Neospora caninum* infections in dogs. *Top. Companion Anim. Med.* 25 170–175. 10.1053/j.tcam.2010.07.005 20937501

[B31] Mota C. M. Oliveira A. C. M. Davoli-Ferreira M. Silva M. V. Santiago F. M. Nadipuram S. M. (2016). *Neospora caninum* activates p38 MAPK as an evasion mechanism against innate immunity. *Front. Microbiol.* 7:1456 10.3389/fmicb.2016.01456 PMC5020094 27679624

[B32] Nolan S. J. Romano J. D. Luechtefeld T. Coppens I. (2015). *Neospora caninum* recruits host cell structures to its parasitophorous vacuole and salvages lipids from organelles. *Eukaryot. Cell* 14 454–473. 10.1128/EC.00262-14 25750213 PMC4421005

[B33] Pan H. Na Y. Xia C. Jing L. Daoyu Y. Qun L. (2014). First isolation of *Neospora caninum* from blood of a naturally infected adult dairy cow in Beijing, China. *J. Parasitol.* 100 812–816. 10.1645/14-498.1 24945568

[B34] Pastor-Fernández I. Regidor-Cerrillo J. Jiménez-Ruiz E. Álvarez-García G. Marugán-Hernández V. Hemphill A. (2016). Characterization of the *Neospora caninum* NcROP40 and NcROP2Fam-1 rhoptry proteins during the tachyzoite lytic cycle. *Parasitology* 143 97–113. 10.1017/S0031182015001511 26521890

[B35] Payne T. M. Payne A. J. Knoll L. J. (2011). A *Toxoplasma gondii* mutant highlights the importance of translational regulation in the apicoplast during animal infection. *Mol. Microbiol.* 82 1204–1216. 10.1111/j.1365-2958.2011.07879.x 22059956 PMC4348008

[B36] Pereira G. D. Regidorcerrillo J. Collantesfernández E. Aguadomartínez A. Del P. I. Minguijón E. (2010). Pathogenic characterization in mice of *Neospora caninum* isolates obtained from asymptomatic calves. *Parasitology* 137 1057–1068. 10.1017/S0031182009991855 20233488

[B37] Plattner F. Yarovinsky F. Romero S. Didry D. Carlier M. F. Sher A. (2008). Toxoplasma profilin is essential for host cell invasion and TLR11-dependent induction of an interleukin-12 response. *Cell Host Microbe* 3 77–87. 10.1016/j.chom.2008.01.001 18312842

[B38] Radke J. B. Lucas O. De Silva E. K. Ma Y. Sullivan W. J. Weiss L. M. (2013). ApiAP2 transcription factor restricts development of the Toxoplasma tissue cyst. *Proc. Natl. Acad. Sci. U.S.A.* 110 6871–6876. 10.1073/pnas.1300059110 23572590 PMC3637731

[B39] Reese M. L. Boothroyd J. C. (2011). A conserved non-canonical motif in the pseudoactive site of the ROP5 pseudokinase domain mediates its effect on Toxoplasma virulence. *J. Biol. Chem.* 286 29366–29375. 10.1074/jbc.M111.253435 21708941 PMC3190742

[B40] Regidorcerrillo J. Gómezbautista M. Del P. I. Jiménezruiz E. Aduriz G. Ortegamora L. M. (2010). Influence of *Neospora caninum* intra-specific variability in the outcome of infection in a pregnant BALB/c mouse model. *Vet. Res.* 41 472–476. 10.1051/vetres/2010024 PMC2878169 20416260

[B41] Reid A. J. Vermont S. J. Cotton J. A. Harris D. Hill-Cawthorne G. A. Knen-Waisman S. (2012). Comparative genomics of the apicomplexan parasites *Toxoplasma gondii* and *Neospora caninum*: coccidia differing in host range and transmission strategy. *PLoS Pathog.* 8:e1002567 10.1371/journal.ppat.1002567 PMC3310773 22457617

[B42] Roos D. S. Donald R. G. Morrissette N. S. Moulton A. L. (1994). Molecular tools for genetic dissection of the protozoan parasite *Toxoplasma gondii*. *Method Cell Biol.* 45 27–63. 10.1016/S0091-679X(08)61845-2 7707991

[B43] Saeij J. P. J. Boyle J. P. Coller S. Taylor S. Sibley L. D. Brooke-Powell E. T. (2006). Polymorphic secreted kinases are key virulence factors in toxoplasmosis. *Science* 314 1780–1783. 10.1126/science.1133690 17170306 PMC2646183

[B44] Sandberg A. Lindell G. Källström B. N. Branca R. M. Danielsson K. G. Dahlberg M. (2012). Tumor proteomics by multivariate analysis on individual pathway data for characterization of vulvar cancer phenotypes. *Mol. Cell. Proteom.* 7 M112.016998 10.1074/mcp.M112.016998 PMC3394958 22499770

[B45] Shen B. Brown K. M. Lee T. D. Sibley L. D. (2014). Efficient gene disruption in diverse strains of *Toxoplasma gondii* using CRISPR/CAS9. *mBio* 5:e1114-14 10.1128/mBio.01114-14 PMC4030483 24825012

[B46] Shwab E. K. Jiang T. Pena H. F. J. Gennari S. M. Dubey J. P. Su C. (2015). The ROP18 and ROP5 gene allele types are highly predictive of virulence in mice across globally distributed strains of *Toxoplasma gondii*. *Int. J. Parasitol.* 46 141–146. 10.1016/j.ijpara.2015.10.005 26699401

[B47] Sinai A. P. Joiner K. A. (2001). The *Toxoplasma gondii* protein ROP2 mediates host organelle association with the parasitophorous vacuole membrane. *J. Cell Biol.* 154 95–108. 10.1083/jcb.200101073 11448993 PMC2196872

[B48] Soldati D. Meissner M. (2004). Toxoplasma as a novel system for motility. *Curr. Opin. Cell Biol.* 16 32–40. 10.1016/j.ceb.2003.11.013 15037302

[B49] Su C. Evans D. Cole R. H. Kissinger J. C. Ajioka J. W. Sibley L. D. (2003). Recent expansion of Toxoplasma through enhanced oral transmission. *Science* 299 414–416. 10.1126/science.1078035 12532022

[B50] Tao L. Hui W. Jing L. Huizhu N. Qun L. (2014). ROP18 is a key factor responsible for virulence difference between *Toxoplasma gondii* and *Neospora caninum*. *PLoS ONE* 9:e99744 10.1371/journal.pone.0099744 PMC4057265 24927100

[B51] Taylor S. Barragan A. Su C. Fux B. Fentress S. J. Tang K. (2006). A secreted serine-threonine kinase determines virulence in the eukaryotic pathogen *Toxoplasma gondii*. *Science* 314 1776–1780. 10.1126/science.1133643 17170305

[B52] Tilley M. Fichera M. E. Jerome M. E. Roos D. S. White M. W. (1997). *Toxoplasma gondii* sporozoites form a transient parasitophorous vacuole that is impermeable and contains only a subset of dense-granule proteins. *Infect. Immun.* 65 4598–4605. 9353039 10.1128/iai.65.11.4598-4605.1997 PMC175660

[B53] Vemulapalli R. Sanakkayala N. Gulani J. Schurig G. G. Boyle S. M. Lindsay D. S. (2007). Reduced cerebral infection of *Neospora caninum* in BALB/c mice vaccinated with recombinant *Brucella* abortus RB51 strains expressing *N. caninum SRS*2 and GRA7 proteins. *Vet. Parasitol.* 148 219–230. 10.1016/j.vetpar.2007.06.029 17651896

[B54] Walker R. Gissot M. Croken M. M. Huot L. Hot D. Kim K. (2013a). TheToxoplasma nuclear factor TgAP2XI-4 controls bradyzoite gene expression and cyst formation. *Mol. Microbiol.* 87 641–655. 10.1111/mmi.12121 23240624 PMC3556193

[B55] Walker R. Gissot M. Huot L. Alayi T. D. Hot D. Marot G. (2013b). Toxoplasma transcription factor TgAP2XI-5 regulates the expression of genes involved in parasite virulence and host invasion. *J. Biol. Chem.* 43 31127–31138. 10.1074/jbc.M113.486589 PMC3829425 24025328

[B56] Walzer K. A. Adomako-Ankomah Y. Dam R. A. Herrmann D. C. Schares G. Dubey J. P. (2013). Hammondia hammondi, an avirulent relative of *Toxoplasma gondii*, has functional orthologs of known *T. gondii* virulence genes. *Proc. Natl. Acad. Sci. U.S.A.* 110 7446–7451. 10.1073/pnas.1304322110 23589877 PMC3645575

[B57] Wang J. Tang D. Li W. Xu J. Liu Q. Liu J. (2017). A new microneme protein of *Neospora caninum*, NcMIC8 is involved in host cell invasion. *Exp. Parasitol.* 175 21–27. 10.1016/j.exppara.2017.01.004 28130119

[B58] Williams M. J. Alonso H. Enciso M. Egarter S. Sheiner L. Meissner M. (2015). Two essential light chains regulate the MyoA lever arm to promote Toxoplasma gliding motility. *mBio* 6:e00845–15. 10.1128/mBio.00845-15 26374117 PMC4600101

[B59] Yamamoto M. Standley D. M. Takashima S. Saiga H. Okuyama M. Kayama H. (2009). A single polymorphic amino acid on *Toxoplasma gondii* kinase ROP16 determines the direct and strain-specific activation of Stat3. *J. Exp. Med.* 206 2747–2760. 10.1084/jem.20091703 19901082 PMC2806617

[B60] Zhang G. Sugimoto C. Fujisaki K. (2007). Apical membrane antigen 1 is a cross-reactive antigen between *Neospora caninum* and *Toxoplasma gondii*, and the anti-NcAMA1 antibody inhibits host cell invasion by both parasites. *Mol. Biochem. Parasitol.* 151 205–212. 10.1016/j.molbiopara.2006.11.005 17156863

