# Supplementary figures and images for: Rhoptry protein 5 (ROP5) Is a Key Virulence Factor in Neospora caninum
Source: Front Microbiol. 2017 Mar 7;8:370. doi: 10.3389/fmicb.2017.00370 (PMC5340095; doi:10.3389/fmicb.2017.00370)

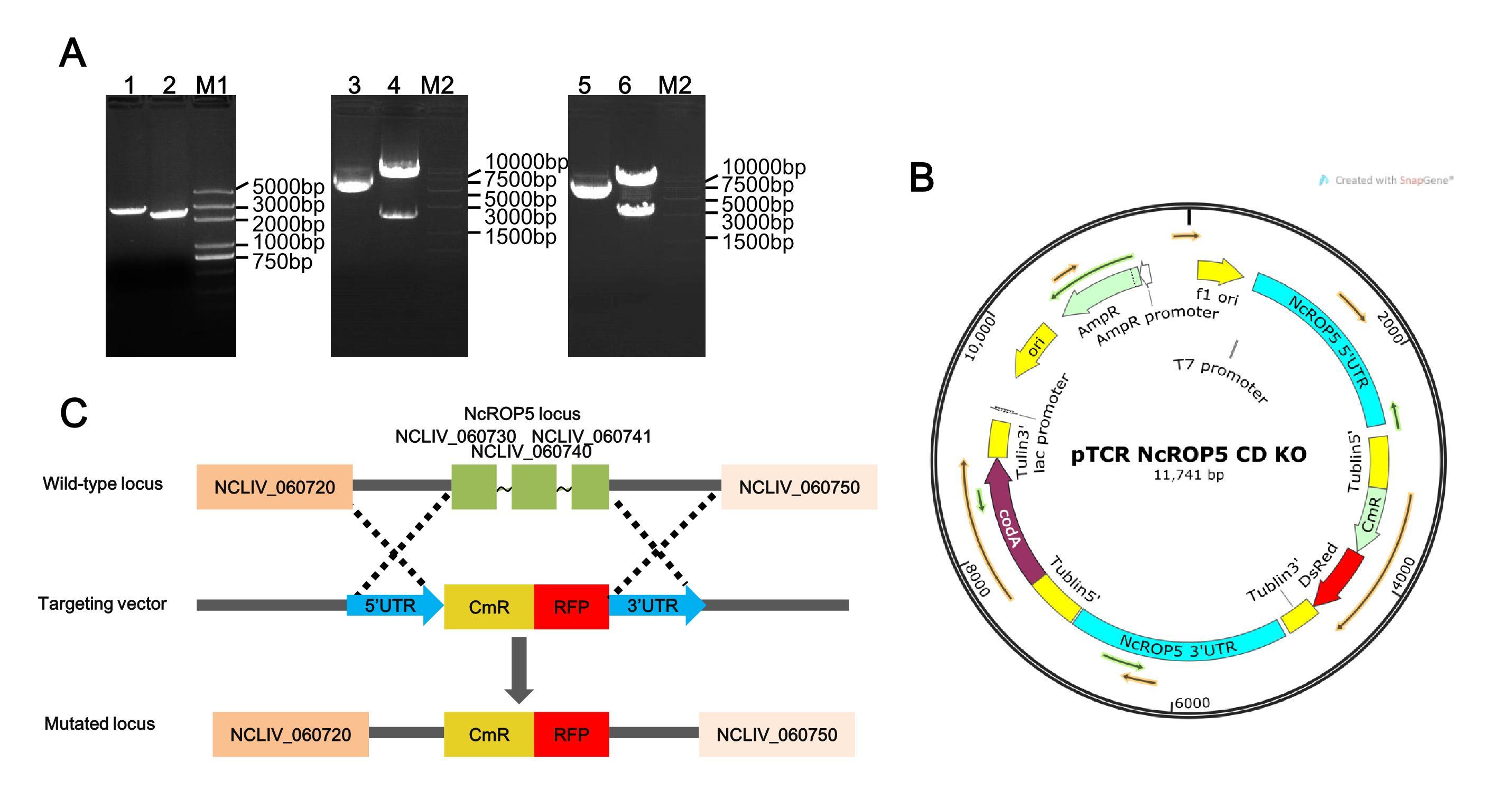

Supplement: FIGURE S1 — Construction of pTCR-NcROP5-CD KO. (A) Products of NcROP5 5′ UTR and 3′ UTR by PCR and identification of pTCR-NcROP5-CD KO by enzyme digestion. 1: NcROP5 5′ UTR; 2: NcROP5 3′ UTR; 3, 5: The knockout plasmid pTCR-NcROP5-CD KO; 4: Double enzyme restriction of pTCR-NcROP5-CD KO for NcROP5 5′ UTR; 6: Double enzyme restriction of pTCR-NcROP5-CD KO for NcROP5 3′ UTR; M1: Trans2K plus DNA marker; M2: Trans15k DNA marker. (B,C) Schemes of the targeted disruption of the NcROP5 gene by double homologous recombination. The pTCR-NcROP5-CD KO plasmid contains the 5′ UTR and 3′ UTR sequences flanking the CmR and RFP marker, which confers resistance to chloramphenicol. [file Image_1.JPEG]

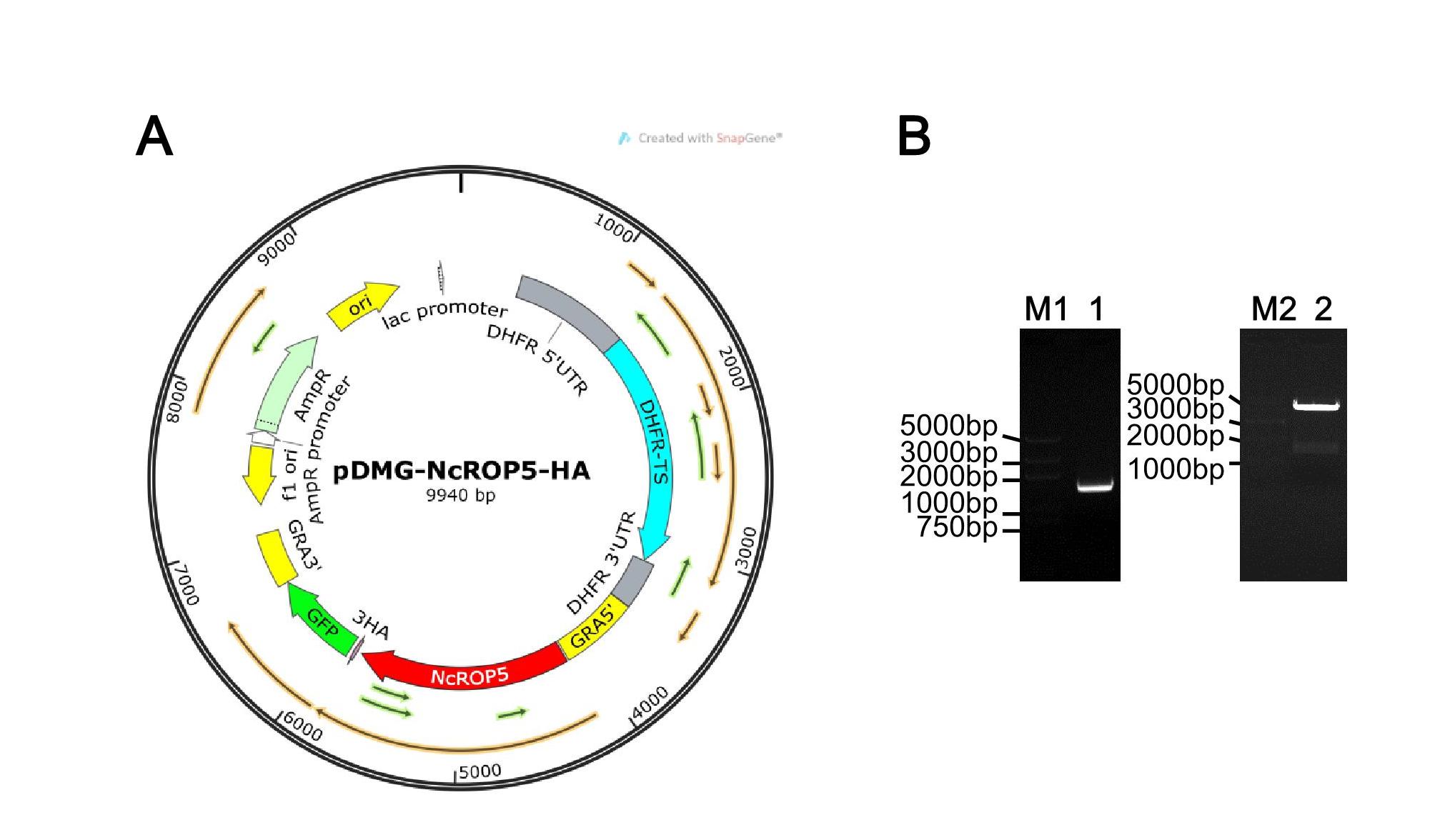

Supplement: FIGURE S2 — Construction of ΔNcROP5 complementary strain, iΔNcROP5. (A) Products of NcROP5A by PCR and identification of the pDMG-NcROP5-HA plasmid by enzyme digestion. 1: NcROP5A coding gene. 2: Double enzyme restricted pDMG-NcROP5-HA. M1: DNA 2000 plus M2: Trans 15K. (B) Diagram of the pDMG-NcROP5-HA construct. [file Image_2.JPEG]

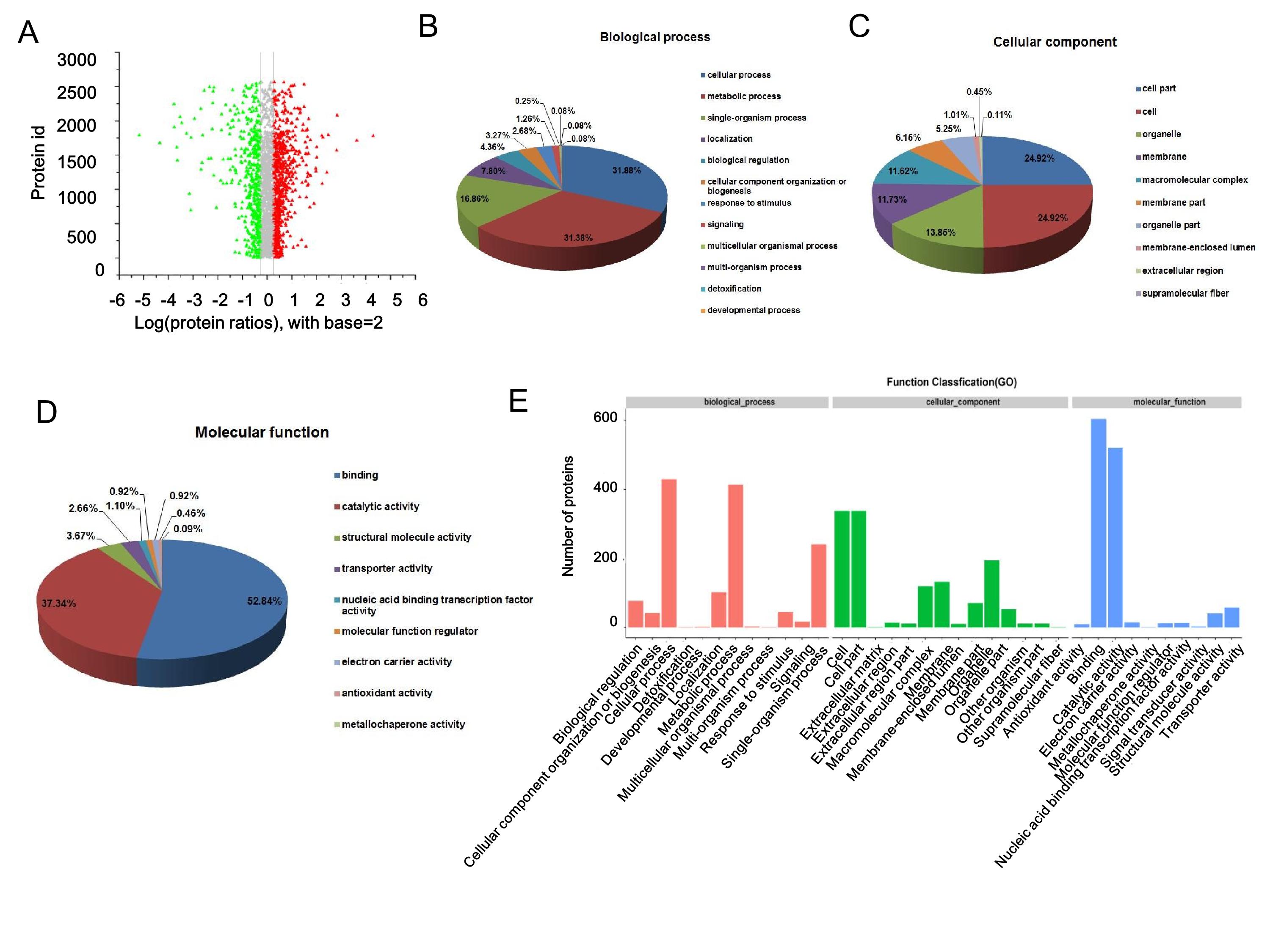

Supplement: FIGURE S3 — Gene ontology analyses of differential proteins in Nc-1 and ΔNcROP5. (A) Differential analysis between Nc-1 and ΔNcROP5. (B) Biological process. Cellular process (31.88%), Metabolic process (31.38%), Single-Organism Process (16.86%), Localization (7.80%), Biological regulation (4.36%), Cellular component organization or biogenesis (3.27%), Response to stimulus (2.68%), Signaling (1.26%), Multicellular organismal process (0.25%), Multi-Organism process (0.08%), Detoxofication (0.08%), Developmental process (0.08%); (C) Cellular component. Cell part (24.92%), Cell (24.92%), Organelle (13.85%), Membrane (11.73%), Macromolecular complex (11.62%), Membrane part (6.15%), Organelle part (5.25%), Membrane-enclosed lumen (1.01%), Extracellular region (0.45%), Supramolecular fiber (0.11%); (D) Molecular function. Binding (52.84%), Catalytic activity (37.34%), Structural molecule activity (3.67%), Transporter activity (2.66%), Nucleic acid binding transcription factor activity (1.10%), Molecular function regulator (0.92%), Electron carrier activity (0.92%), Antioxidant activity (0.46%), Metallochaperone activity (0.09%); (E) Number of proteins involved in biological process, cellular component and molecular function. [file Image_3.JPEG]

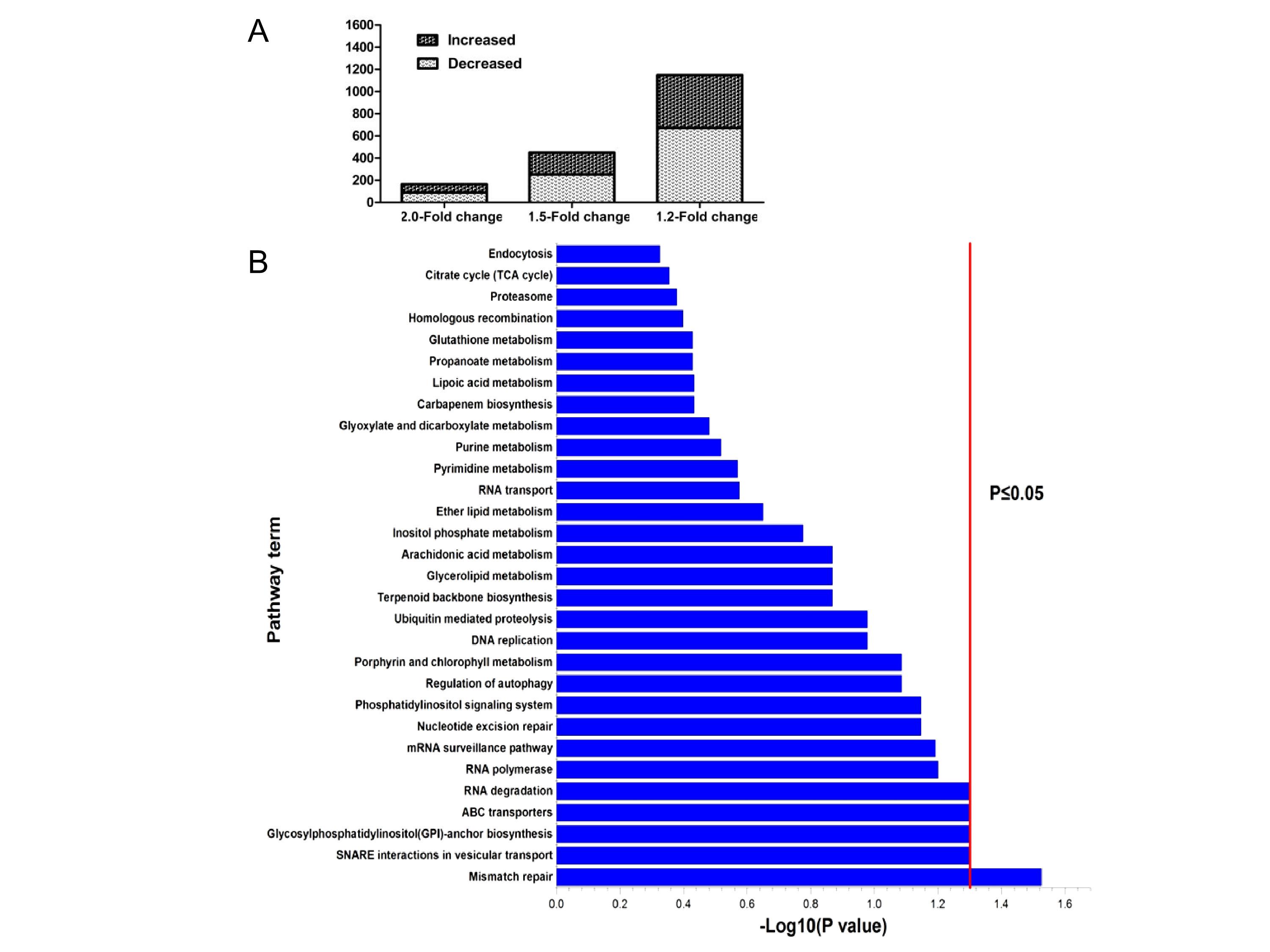

Supplement: FIGURE S4 — The number of differential proteins and classification of enriched pathways in Nc-1 and ΔNcROP5. (A) Increased and decreased proteins are divided into three groups: differentially expressed proteins with a 1.2-, 1.5-, and 2.0-fold difference. (B) Proteins with 1.5-fold difference are categorized by enrichment analysis to 39 pathways. [file Image_4.JPEG]
