# Supplementary material for: Rhoptry protein 5 (ROP5) Is a Key Virulence Factor in Neospora caninum
Source: Front Microbiol. 2017 Mar 7;8:370. doi: 10.3389/fmicb.2017.00370 (PMC5340095; doi:10.3389/fmicb.2017.00370)
Supplement: Supplementary file 5 [file Table_1.DOCX]

**Supplemental Table 1. Oligonucleotides used in this study**

| Primer name | Nucleotide sequence |
| --- | --- |
| F1 | 5′-ggGGTACCTATATGCGGGGGCGTTTGTT-3′ |
| R1 | 5′-ccgCTCGAGTAATGATAATAAGCGGCGTGTGC-3′ |
| F2 | 5′-ccCCCGGGGAGCGCGAGGTCCAATGTCA-3′ |
| R2 | 5′-cggACTAGTCTGCCAGCGCGTATGTTTTCTA-3′ |
| F3 | 5′-cgGATATCAAAATGAAGGCGAGCAGTCCCAAG-3′ |
| R3 | 5′-cgCCTAGGCGTCGCGGCGGCGGGTCCTTGTGCC-3′ |
| F4 | 5′-GCTGTGTCGGCGGCTCTGTG-3′ |
| R4 | 5′-ATCCCGGTGTCGCAAGTCCAAAAG-3′ |
| F5 | 5′- CTCGCAGTCAACCTACGTCTTCT-3′ |
| R5 | 5′- CCCAGTGCGTCCAATCCTGTAAC-3′ |
| ROP5-F | 5′-GCTGTGTCGGCGGCTCTGTGG-3′ |
| ROP5-R | 5′-TGGGCTCTGGGCAATCAACTTCTG-3′ |
| NcActin-F | 5′-GGTTTGCCGGCGCCTTCGTC-3′ |
| NcActin-R | 5′-GGCGGAGCGTCGGGGGATG-3′ |
| RON2-F | 5′- TGCGCCGCCCAACAGACG-3′ |
| RON2-R | 5′- GCCGCCAGAGTGACCCAGGAATAG-3′ |
| RON4-F | 5′-AGGCGGCGAAACTGCAAACTCTC-3′ |
| RON4-R | 5′-CGCCCGTAACTGGTGTCCAC-3′ |
| ROP4-F | 5′-AGTGTGGGCGGGGCTCCTTG-3′ |
| ROP4-R | 5′-CGGTTGGCGGGCTCTTCGTCG-3′ |
| ROP7-F | 5′-TGAAGCGACAGACGTAGCGACAC-3′ |
| ROP7-R | 5′-CGGCAGCGACCAAAAACCTTAG-3′ |
| ROP16-F | 5′-GACACCGCTCGGATGTGAT-3′ |
| ROP16-R | 5′-CTTTACCGTCTCCAAGCCAAT-3′ |
| ROP17-F | 5′-GGGGGATGACGGTTTGTTTTCTGA-3′ |
| ROP17-R | 5′-CCGCGGCTGGGTTCTGTG-3′ |
| GRA2-F | 5′-TCCCCGTGGCGATGATGTTAG-3′ |
| GRA2-R | 5′-GCTTTTGCCGCTGCCTGTTTTAC-3′ |
| GRA6-F | 5′-CCTTCATGGGCGTGCCTCTCAGC-3′ |
| GRA6-R | 5′-GCGCGTTCCTCCGTATCAATCGTC-3′ |
| GRA7-F | 5′-GCAGAAGCCGCACACGACTCA-3′ |
| GRA7-R | 5′-TTTTTACCGGGGATGGACTCTGTT-3′ |
