# Supplementary material for: Rhoptry protein 5 (ROP5) Is a Key Virulence Factor in Neospora caninum
Source: Front Microbiol. 2017 Mar 7;8:370. doi: 10.3389/fmicb.2017.00370 (PMC5340095; doi:10.3389/fmicb.2017.00370)
Supplement: Supplementary file 6 [file Table_2.DOCX]

**Supplemental Table 2. Label-free LC-MS/MS settings**

| Item | Value |
| --- | --- |
| Database | ToxoDB-28_NcaninumLIV_AnnotatedProteins.fasta |
| Enzyme | Trypsin |
| Max Missed Cleavages | 2 |
| Fixed modifications | Carbamidomethyl (C)， |
| Variable modifications | Oxidation (M)，Acetyl （Protein N-term） |
| Peptide Mass Tolerance | ± 15ppm |
| Fragment Mass Tolerance | 20 mmu |
| Peptide confidence | high |
| Peptide length | >4 |

Parameter value: Peptide FDR≤0.01.
