# Supplementary material for: Rhoptry protein 5 (ROP5) Is a Key Virulence Factor in Neospora caninum
Source: Front Microbiol. 2017 Mar 7;8:370. doi: 10.3389/fmicb.2017.00370 (PMC5340095; doi:10.3389/fmicb.2017.00370)
Supplement: Supplementary file 7 [file Table_3.DOCX]

**Supplemental Table 3. The differential proteins of the Nc-1 and ΔNcROP5 strains (Fold changes ≤0.5 or ≥2.0).**

| Accession | Description | ΔNcROP5 v.s. Nc-1 |
| --- | --- | --- |
| NCLIV_0167 | DNA-directed RNA polymerases I, II, and III subunit RPABC1; length=150 aa | 0.0509 |
| NCLIV_027520 | A conserved hypothetical protein; length=3015 aa | 0.0801 |
| NCLIV_038990 | A conserved hypothetical protein; length=522 aa | 0.1404 |
| NCLIV_026230 | DIS3-like exonuclease 1, related; length=1231 aa | 0.1420 |
| NCLIV_016040 | A conserved hypothetical protein; length=1330 aa | 0.1797 |
| NCLIV_045930 | A putative glutaredoxin; length=113 aa | 0.1871 |
| NCLIV_027070 | A conserved hypothetical protein; length=974 aa | 0.2094 |
| NCLIV_028180 | DNA-directed RNA polymerase subunit beta; length=1260 aa | 0.2130 |
| NCLIV_058500 | Putative EF hand domain-containing protein; length=6326 aa | 0.2159 |
| NCLIV_000670 | Putative RNA recognition motif domain-containing protein; length=367 aa | 0.2485 |
| NCLIV_043990 | A conserved hypothetical protein; length=421 aa | 0.2485 |
| NCLIV_042760 | Dehydrogenases with different specificities,related ; length=305 aa | 0.2541 |
| NCLIV_021280 | A conserved hypothetical protein; length=310 aa | 0.2556 |
| NCLIV_060900 | A conserved hypothetical protein; length=1938 aa | 0.2684 |
| NCLIV_028950 | A hypothetical protein; length=2552 aa | 0.2958 |
| NCLIV_013940 | Heat shock protein 17.4, related; length=275 aa | 0.2967 |
| NCLIV_004280 | A hypothetical protein; length=762 aa | 0.3017 |
| NCLIV_009210 | A hypothetical protein; length=3773 aa | 0.3124 |
| NCLIV_009630 | Putative cyclophilin; length=773 aa | 0.3124 |
| NCLIV_013620 | Ubiquitin carboxyl-terminal hydrolase, related; length=414 aa | 0.3128 |
| NCLIV_059380 | SJCHGC06128 protein, related; length=289 aa | 0.3144 |
| NCLIV_028210 | A conserved hypothetical protein; length=424 aa | 0.3195 |
| NCLIV_033100 | Putative prolidase; length=597 aa | 0.3215 |
| NCLIV_004090 | A hypothetical protein; length=378 aa | 0.3228 |
| NCLIV_052980 | A hypothetical protein; length=392 aa | 0.3232 |
| NCLIV_057020 | A conserved hypothetical protein; length=1546 aa | 0.3234 |
| NCLIV_039470 | A conserved hypothetical protein; length=120 aa | 0.3277 |
| NCLIV_041680 | Putative Casein kinase one (CK1) TgCK1b; length=409 aa | 0.3277 |
| NCLIV_016250 | Putative cytidine deaminase; length=417 aa | 0.3328 |
| NCLIV_063180 | Putative ARID/BRIGHT DNA-binding domain-containing protein; length=2830 aa | 0.3340 |
| NCLIV_016900 | A hypothetical protein; length=894 aa | 0.3359 |
| NCLIV_002590 | Phthalate dioxygenase reductase subunit, related; length=681 aa | 0.3363 |
| NCLIV_0234 | Glutamine amidotransferase, SNO family domain-containing protein; length=279 aa | 0.3383 |
| NCLIV_065310 | A conserved hypothetical protein; length=125 aa | 0.3388 |
| NCLIV_051920 | A conserved hypothetical protein; length=944 aa | 0.3429 |
| NCLIV_005020 | Putative PUA domain-containing, cell cycle regulator protein; length=179 aa | 0.3550 |
| NCLIV_046320 | A conserved hypothetical protein; length=922 aa | 0.3550 |
| NCLIV_026250 | NADPH:adrenodoxin oxidoreductase FprB, related; length=661 aa | 0.3643 |
| NCLIV_000530 | 3-oxoacyl-(Acyl-carrier-protein) synthase II,related; length=579 aa | 0.3651 |
| NCLIV_019460 | A hypothetical protein; length=416 aa | 0.3700 |
| NCLIV_069400 | A conserved hypothetical protein; length=208 aa | 0.3727 |
| NCLIV_025280 | A conserved hypothetical protein; length=294 aa | 0.3759 |
| NCLIV_038100 | A hypothetical protein; length=351 aa | 0.3783 |
| NCLIV_031320 | A conserved hypothetical protein; length=1583 aa | 0.3803 |
| NCLIV_020920 | A conserved hypothetical protein; length=2250 aa | 0.3951 |
| NCLIV_042170 | Phd finger protein BR140/LIN-49, related; length=4543 aa | 0.3962 |
| NCLIV_041640 | A conserved hypothetical protein; length=339 aa | 0.3994 |
| NCLIV_030410 | A conserved hypothetical protein; length=462 aa | 0.3994 |
| NCLIV_045220 | A hypothetical protein; length=398 aa | 0.4014 |
| NCLIV_034650 | A conserved hypothetical protein; length=306 aa | 0.4047 |
| NCLIV_065020 | Putative actin-like protein 3b; length=663 aa | 0.4076 |
| NCLIV_036630 | Putative 14-3-3 protein; length=547 aa | 0.4096 |
| NCLIV_051870 | Putative DNA repair enzyme; length=474 aa | 0.4163 |
| NCLIV_067140 | Myosin, related; length=1941 aa | 0.4228 |
| NCLIV_046200 | A hypothetical protein; length=1319 aa | 0.4260 |
| NCLIV_025650 | A conserved hypothetical protein; length=1161 aa | 0.4317 |
| NCLIV_003620 | Putative KH domain-containing protein; length=1431 aa | 0.4357 |
| NCLIV_022990 | A conserved hypothetical protein; length=299 aa | 0.4402 |
| NCLIV_054960 | Putative vacuolar ATP synthase subunit F; length=127 aa | 0.4412 |
| NCLIV_063490 | A conserved hypothetical protein; length=520 aa | 0.4484 |
| NCLIV_037070 | A conserved hypothetical protein; length=245 aa | 0.4506 |
| NCLIV_032950 | Putative deoxyuridine 5'-triphosphate nucleotidohydrolase; length=188 aa | 0.4536 |
| NCLIV_005750 | A conserved hypothetical protein; length=778 aa | 0.4542 |
| NCLIV_034950 | Putative poly(A)+ RNA export protein; length=340 aa | 0.4564 |
| NCLIV_014350 | A hypothetical protein; length=114 aa | 0.4584 |
| NCLIV_070150 | A conserved hypothetical protein; length=521 aa | 0.4585 |
| NCLIV_056570 | Collagen alpha-1(III) chain (Precursor), related; length=2814 aa | 0.4587 |
| NCLIV_048410 | A conserved hypothetical protein; length=154 aa | 0.4652 |
| NCLIV_016300 | A conserved hypothetical protein; length=626 aa | 0.4675 |
| NCLIV_049890 | Putative ubiquitin-conjugating enzyme; length=293 aa | 0.4686 |
| NCLIV_009310 | Putative trafficking protein particle complex subunit 3; length=187 aa | 0.4720 |
| NCLIV_061350 | Putative glutathione reductase; length=635 aa | 0.4774 |
| NCLIV_045460 | Mitochondrial presequence protease (Precursor), related; length=1131 aa | 0.4792 |
| NCLIV_020380 | A conserved hypothetical protein; length=654 aa | 0.4792 |
| NCLIV_010630 | cDNA FLJ53078, highly similar to Splicing factor, arginine/serine-rich 1, related; length=448 aa | 0.4809 |
| NCLIV_054020 | A conserved hypothetical protein; length=3094 aa | 0.4841 |
| NCLIV_009140 | A conserved hypothetical protein; length=372 aa | 0.4841 |
| NCLIV_023550 | Putative hydroxyacylglutathione hydrolase; length=266 aa | 0.4851 |
| NCLIV_025480 | Putative kelch motif domain-containing protein; length=804 aa | 0.4893 |
| NCLIV_031640 | Putative DNA-directed RNA polymerase II; length=130 aa | 0.4893 |
| NCLIV_067580 | Unspecified product; length=400 aa | 0.4899 |
| NCLIV_037490 | Unspecified product; length=444 aa | 0.4915 |
| NCLIV_029590 | A hypothetical protein; length=457 aa | 0.4937 |
| NCLIV_030890 | Putative high molecular mass nuclear antigen; length=918 aa | 0.4952 |
| NCLIV_046160 | A conserved hypothetical protein; length=758 aa | 0.4953 |
| NCLIV_040010 | Putative alpha-1 type II collagen; length=1567 aa | 0.4962 |
| NCLIV_036200 | Putative cyclophilin; length=198 aa | 0.4962 |
| NCLIV_016700 | A conserved hypothetical protein; length=292 aa | 0.4997 |
| NCLIV_029290 | A hypothetical protein; length=1203 aa | 0.4999 |
| NCLIV_040650 | A conserved hypothetical protein; length=2379 aa | 2.0093 |
| NCLIV_023110 | A hypothetical protein; length=119 aa | 2.0331 |
| NCLIV_039500 | A hypothetical protein; length=4250 aa | 2.0342 |
| NCLIV_013070 | A conserved hypothetical protein; length=423 aa | 2.0386 |
| NCLIV_065140 | Putative endonuclease/exonuclease/phosphatase domain-containing protein; length=507 aa | 2.0538 |
| NCLIV_016760 | A hypothetical protein; length=205 aa | 2.0573 |
| NCLIV_058470 | A hypothetical protein; length=768 aa | 2.0707 |
| NCLIV_003470 | Putative thrombospondin type 1 domain-containing protein; length=822 aa | 2.0738 |
| NCLIV_026710 | A conserved hypothetical protein; length=1017 aa | 2.0919 |
| NCLIV_020880 | A hypothetical protein; length=133 aa | 2.1299 |
| NCLIV_047680 | A conserved hypothetical protein; length=1055 aa | 2.1299 |
| NCLIV_046260 | Iron regulatory protein-like protein, related; length=968 aa | 2.1859 |
| NCLIV_019450 | A hypothetical protein; length=223 aa | 2.2118 |
| NCLIV_064260 | Putative WD domain-containing protein; length=3128 aa | 2.2186 |
| NCLIV_060500 | A conserved hypothetical protein; length=550 aa | 2.2313 |
| NCLIV_034930 | A conserved hypothetical protein; length=565 aa | 2.2329 |
| NCLIV_059950 | Unspecified product; length=779 aa | 2.3235 |
| NCLIV_025450 | Putative elongation factor Tu; length=488 aa | 2.3465 |
| NCLIV_060160 | Tgd057, related; length=193 aa | 2.3961 |
| NCLIV_039520 | A conserved hypothetical protein; length=971 aa | 2.4203 |
| NCLIV_012680 | A conserved hypothetical protein; length=4607 aa | 2.4288 |
| NCLIV_016840 | AFL091Wp, related; length=324 aa | 2.4342 |
| NCLIV_059500 | Putative nucleotidase; length=713 aa | 2.4494 |
| NCLIV_004580 | A hypothetical protein; length=3520 aa | 2.4494 |
| NCLIV_029810 | Tcc1a22.4, related; length=516 aa | 2.4576 |
| NCLIV_066770 | Putative seryl-tRNA synthetase; length=480 aa | 2.4627 |
| NCLIV_065500 | A conserved hypothetical protein; length=311 aa | 2.4676 |
| NCLIV_041460 | putative adaptin N terminal region domain-containing protein; length=1211 aa | 2.5102 |
| NCLIV_007930 | A hypothetical protein; length=359 aa | 2.5863 |
| NCLIV_006160 | Multi-pass transmembrane protein, related; length=1645 aa | 2.6063 |
| NCLIV_006800 | A conserved hypothetical protein; length=785 aa | 2.6187 |
| NCLIV_044900 | A hypothetical protein; length=6442 aa | 2.6328 |
| NCLIV_068960 | A hypothetical protein; length=338 aa | 2.6624 |
| NCLIV_061390 | A conserved hypothetical protein; length=315 aa | 2.6904 |
| NCLIV_054500 | A hypothetical protein; length=134 aa | 2.7250 |
| NCLIV_002020 | A hypothetical protein; length=642 aa | 2.7891 |
| NCLIV_036760 | A hypothetical protein; length=1123 aa | 2.8103 |
| NCLIV_042330 | A conserved hypothetical protein; length=165 aa | 2.8145 |
| NCLIV_000260 | A conserved hypothetical protein; length=1675 aa | 2.8190 |
| NCLIV_039990 | A conserved hypothetical protein; length=348 aa | 2.8620 |
| NCLIV_006420 | A conserved hypothetical protein; length=872 aa | 3.0047 |
| NCLIV_036080 | A conserved hypothetical protein; length=1186 aa | 3.0140 |
| NCLIV_004390 | p23, related; length=237 aa | 3.1169 |
| NCLIV_042190 | A conserved hypothetical protein; length=727 aa | 3.1948 |
| NCLIV_039710 | Multitransmembrane protein with signal peptide and GMGPP repeat at C-terminus, related; length=376 aa | 3.4353 |
| NCLIV_012890 | A hypothetical protein; length=7289 aa | 3.4539 |
| NCLIV_004260 | A hypothetical protein; length=761 aa | 3.6044 |
| NCLIV_045570 | A hypothetical protein; length=624 aa | 3.6132 |
| NCLIV_012090 | Putative ATP-dependent protease ATP-binding subunit; length=670 aa | 3.6863 |
| NCLIV_022270 | Unspecified product; length=691 aa | 4.1672 |
| NCLIV_007590 | Putative actin-like family protein ARP4a; length=757 aa | 4.4534 |
| NCLIV_043140 | Putative aspartate carbamoyltransferase; length=363 aa | 4.4728 |
| NCLIV_016050 | A hypothetical protein; length=2643 aa | 4.5640 |
| NCLIV_054760 | A conserved hypothetical protein; length=571 aa | 4.6148 |
| NCLIV_003960 | A hypothetical protein; length=234 aa | 4.6754 |
| NCLIV_024040 | Aminotransferase, related; length=594 aa | 4.8332 |
| NCLIV_003060 | Putative capping protein alpha-like subunit; length=426 aa | 5.0585 |
| NCLIV_033790 | Putative replication factor c; length=403 aa | 5.1808 |
| NCLIV_029920 | Receptor expression-enhancing protein 3, related; length=216 aa | 5.8572 |
| NCLIV_036380 | A conserved hypothetical protein; length=373 aa | 6.1439 |
| NCLIV_032930 | Unspecified product; length=2675 aa | 6.2122 |
| NCLIV_054830 | Unspecified product; length=336 aa | 6.3897 |
| NCLIV_057420 | Putative WW domain-containing protein; length=2422 aa | 6.4457 |
| NCLIV_045660 | A conserved hypothetical protein; length=3561 aa | 7.7117 |
| NCLIV_065560 | A hypothetical protein; length=559 aa | 8.4761 |
| NCLIV_047110 | A conserved hypothetical protein; length=1509 aa | 8.5196 |
| NCLIV_011110 | Putative DEAH-box RNA/DNA helicase; length=2254 aa | 8.8745 |
| NCLIV_063730 | Putative thrombospondin type 1 domain-containing protein; length=2616 aa | 9.6352 |
| NCLIV_023520 | A hypothetical protein; length=696 aa | 11.0367 |
| NCLIV_045280 | A conserved hypothetical protein; length=2133 aa | 11.5369 |
| NCLIV_062590 | A conserved hypothetical protein; length=545 aa | 12.4676 |
| NCLIV_006460 | Putative beta-tubulin cofactor d; length=2024 aa | 12.6462 |
| NCLIV_048310 | A hypothetical protein; length=1120 aa | 13.6076 |
| NCLIV_063920 | Unspecified product; length=3991 aa | 13.6921 |
| NCLIV_005630 | A conserved hypothetical protein; length=2520 aa | 20.4114 |
| NCLIV_025960 | Putative nucleolar GTP-binding protein; length=718 aa | 36.3639 |
