# Supplementary material for: Rhoptry protein 5 (ROP5) Is a Key Virulence Factor in Neospora caninum
Source: Front Microbiol. 2017 Mar 7;8:370. doi: 10.3389/fmicb.2017.00370 (PMC5340095; doi:10.3389/fmicb.2017.00370)
Supplement: Supplementary file 8 [file Table_4.DOCX]

**Supplemental Table 4. Differentially expressed proteins found to be enriched in 5 pathways in Nc-1 and ΔNcROP5 (P≤0.05)**

| KEGG pathway | ID | Input number | Background number | P-value | Gene name |
| --- | --- | --- | --- | --- | --- |
| Mismatch repair | ko03430 | 13 | 15 | 0.0298 | NCLIV_042630, NCLIV_050990, NCLIV_026280, NCLIV_011880, NCLIV_065280, NCLIV_013120, NCLIV_033790, NCLIV_049750, NCLIV_050400, NCLIV_021770, NCLIV_007620, NCLIV_033420, NCLIV_003210 |
| SNARE interactions in vesicular transport | ko04130 | 6 | 6 | 0.0497 | NCLIV_024210, NCLIV_010850, NCLIV_041710, NCLIV_009810, NCLIV_042570, NCLIV_034280 |
| Glycosylphosp-hatidylinositol (GPI)-anchor biosynthesis | ko00563 | 6 | 6 | 0.0497 | NCLIV_028260, NCLIV_060640, NCLIV_004260, NCLIV_010770, NCLIV_068300, NCLIV_007520 |
| ABC transporters | ko02010 | 6 | 6 | 0.0497 | NCLIV_028260, NCLIV_060640, NCLIV_004260, NCLIV_010770, NCLIV_068300, NCLIV_007520 |
| RNA degradation | ko03018 | 25 | 33 | 0.0497 | NCLIV_024210, NCLIV_010850, NCLIV_041710, NCLIV_009810, NCLIV_042570, NCLIV_034280, NCLIV_026230, NCLIV_037490, NCLIV_013510, NCLIV_014710, NCLIV_068900, NCLIV_070110, NCLIV_066840, NCLIV_009510, NCLIV_065210, NCLIV_065170, NCLIV_017830, NCLIV_016800, NCLIV_048140, NCLIV_056360, NCLIV_047860, NCLIV_001370, NCLIV_029810, NCLIV_016050, NCLIV_034680, NCLIV_010510, NCLIV_063640, NCLIV_033510, NCLIV_045720, NCLIV_017360, NCLIV_014070 |
